# Defective brown adipose tissue thermogenesis and impaired glucose metabolism in mice lacking Letmd1

**DOI:** 10.1016/j.celrep.2021.110104

**Published:** 2021-12-14

**Authors:** Kyung-Mi Choi, Jung Hak Kim, Xiangmudong Kong, Meltem Isik, Jin Zhang, Hee-Woong Lim, John C. Yoon

**Affiliations:** 1 Division of Endocrinology, Department of Internal Medicine, University of California Davis School of Medicine, Davis, CA 95616, USA; 2 Department of Surgical and Radiological Sciences, University of California Davis School of Veterinary Medicine, Davis, CA 95616, USA; 3 Vor Biopharma, Cambridge, MA 02140, USA; 4 Division of Biomedical Informatics, Cincinnati Children’s Hospital Medical Center; Department of Pediatrics, University of Cincinnati College of Medicine, Cincinnati, OH 45229, USA; 5 Lead contact

## Abstract

Manipulation of energy-dissipating adipocytes has the potential to produce metabolic benefits. To this end, it is valuable to understand the mechanisms controlling the generation and function of thermogenic fat. Here, we identify Letm1 domain containing 1 (Letmd1) as a regulator of brown fat formation and function. The expression of Letmd1 is induced in brown fat by cold exposure and by β-adrenergic activation. Letmd1-deficient mice exhibit severe cold intolerance concomitant with abnormal brown fat morphology, reduced thermogenic gene expression, and low mitochondrial content. The null mice exhibit impaired β3-adrenoreceptor-dependent thermogenesis and are prone to diet-induced obesity and defective glucose disposal. Letmd1 was previously described as a mitochondrial protein, and we find that it also localizes to the nucleus and interacts with the transcriptional coregulator and chromatin remodeler Brg1/Smarca4, thus providing a way to impact thermogenic gene expression. Our study uncovers a role for Letmd1 as a key regulatory component of adaptive thermogenesis.

## In brief

Choi et al. report that Letmd1 deficiency in mice abolishes β3-adrenoreceptor-dependent energy expenditure and impairs systemic glucose metabolism. Because the expression of Letmd1 correlates with adipocyte thermogenesis, these findings suggest that Letmd1 is a regulatory component of the physiological thermogenic response.

## Graphical Abstract

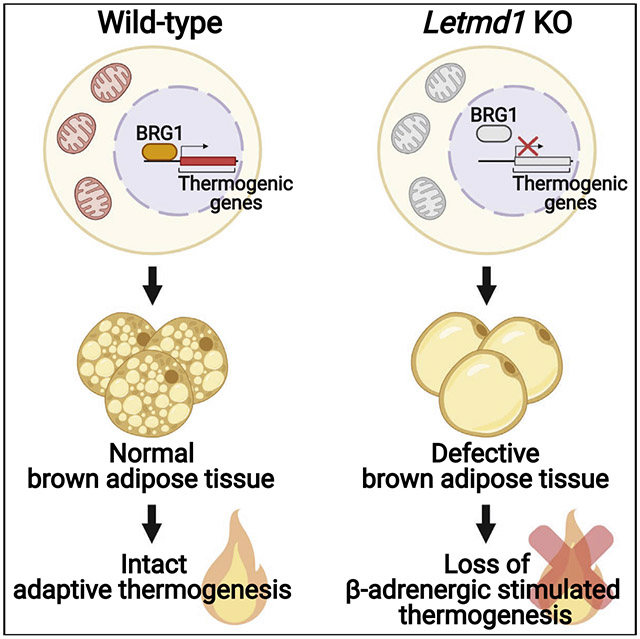

## INTRODUCTION

Animals continually assess their own energy status and use that information to modify food-seeking behavior and the level of energy expenditure. The control mechanisms of energy balance have evolved primarily to deal with energy deficiency, and they may protect less well against conditions of chronic energy overload, which has become prevalent in human society (Hales et al., 2015). In essence, obesity is a consequence of a sustained positive energy balance. Enhancing thermogenesis to increase energy expenditure is thus a rational strategy to combat obesity and associated complications. Recent data indicate that adult humans possess more extensive cold-activated thermogenic adipose tissue than previously estimated (Cypess et al., 2009; Leitner et al., 2017; van Marken Lichtenbelt et al., 2009; Virtanen et al., 2009). Thermogenic fat activity correlates negatively with obesity in humans (Heinitz et al., 2020; Leitner et al., 2017; van Marken Lichtenbelt et al., 2009) and individuals with detectable thermogenic fat have lower odds of developing type 2 diabetes and heart disease (Becher et al., 2021). In addition, inducible thermogenic fat cells can be recruited by a variety of stimuli including chronic cold exposure, bariatric surgery, cancer cachexia, and burn-induced cachexia (Kajimura et al., 2015; van der Lans et al., 2013). Developing new approaches to increase the abundance or activity of thermogenic fat can potentially lead to metabolic benefits. In this sense, understanding the mechanisms of thermogenic fat development and function is essential.

The constitutive and inducible thermogenic fat, referred to as brown and beige (or brite) fat, respectively, differ in their origins (Sanchez-Gurmaches et al., 2016; Shapira and Seale, 2019). Interscapular brown adipocytes arise from *Pax7*^+^/*Myf5*^+^ precursor cells, whereas beige adipocytes come from *Pax7*^−^/*Myf*^−-^ precursor cells (Sanchez-Gurmaches et al., 2016). Brown adipocytes are found in dedicated brown adipose tissue (BAT) depots, and beige adipocytes originate from within non-thermogenic white adipose tissue (WAT). The thermogenic capacity of BAT is primarily mediated through uncoupling protein 1 (UCP1), an inner mitochondrial membrane protein that dissipates the proton gradient upon activation. UCP1 is expressed in both brown and beige fat but absent in white fat. Beige adipocytes also use UCP1-independent thermogenic mechanisms that are currently being actively investigated (Ikeda et al., 2017; Kazak et al., 2017). Certain thermogenesis-related genes such as Ucp1, Cidea, Dio2, and PGC1α are expressed at a higher level in BAT than that in WAT, and a number of transcriptional and epigenetic regulators have been implicated in controlling the program of BAT differentiation and thermogenic gene expression (Gulyaeva et al., 2019; Inagaki et al., 2016; Shapira and Seale, 2019). Prdm16 is a transcriptional coregulator that partners with a variety of transcription factors and cofactors such as C/EBPβ, PGC1α, and PPARγ to increase BAT gene expression (Seale et al., 2007). It is dispensable for BAT development but is necessary for the maintenance of brown adipocyte identity later in life (Harms et al., 2014). The expression of Prdm16 in BAT is not regulated by cold (Seale et al., 2007), suggesting the involvement of other regulatory proteins in cold adaptation. Ebf2 is a transcription factor required for BAT development (Rajakumari et al., 2013). This protein has been reported to recruit PPARγ to the Prdm16 promoter and associate with the chromatin remodeler Brg1 that opens the chromatin for transcription (Shapira et al., 2017). Ebf2 is also expressed in tissues such as the brain and blood cells (Chuang et al., 2011; Kieslinger et al., 2010), and what controls or initiates Ebf2 expression in BAT is not known. PGC1α, another coregulator, controls mitochondrial biogenesis but PGC1α null adipose tissue displays only a modest thermogenic defect (Arany et al., 2005; Kleiner et al., 2012). Other regulators such as C/EBPβ and PPARγ are prominent players in the general adipogenesis program without specificity for the brown adipocyte lineage (Gulyaeva et al., 2019; Park et al., 2012). It has been proposed that the BAT-specific gene transcriptional program is established by changes in the chromatin structure that results in differential recruitment of transcription factors to BAT-specific target gene promoters (Inagaki et al., 2016). We still have an incomplete understanding of the molecules and pathways that govern how brown fat and beige fat cells are formed and how they function, limiting our ability to fully exploit adipose tissue thermogenesis for therapeutic benefits.

In an effort to make progress toward this eventual goal of manipulating energy expenditure, we sought to identify regulators of thermogenic fat development and function. We focused on genes that are differentially expressed in thermogenic fat relative to non-thermogenic fat, both in humans and mice, and also undergo regulation during the recruitment of inducible thermogenic fat. Here, we report that Letm1 domain containing 1 (Letmd1) is a key regulator of thermogenic fat formation and function. Previously described as a mitochondrial outer membrane protein (Cho et al., 2007; Ha et al., 2010), Letmd1 also localizes to the nucleus and regulates thermogenic fat gene expression. The expression of Letmd1 itself is induced by cold stimuli and β-adrenergic agonists and is selective for BAT, while being essentially undetectable in WAT. The loss of Letmd1 in mice results in abnormal brown fat morphology, decreased thermogenic gene and protein expression, and profoundly impaired adaptive thermogenesis, without apparent effects on growth. We find that a target of Letmd1 is the chromatin remodeler Brg1, which is necessary for brown fat function and thermogenic gene expression, raising the possibility that Letmd1 may confer tissue specificity during the course of the thermogenic gene transcription program. Letmd1 serves to link β-adrenergic stimulation to the regulation of thermogenesis and represents a potential therapeutic target.

## RESULTS

### Letmd1 expression is induced in brown and white fat by cold stress and β-adrenergic signaling

**Figure 1.**
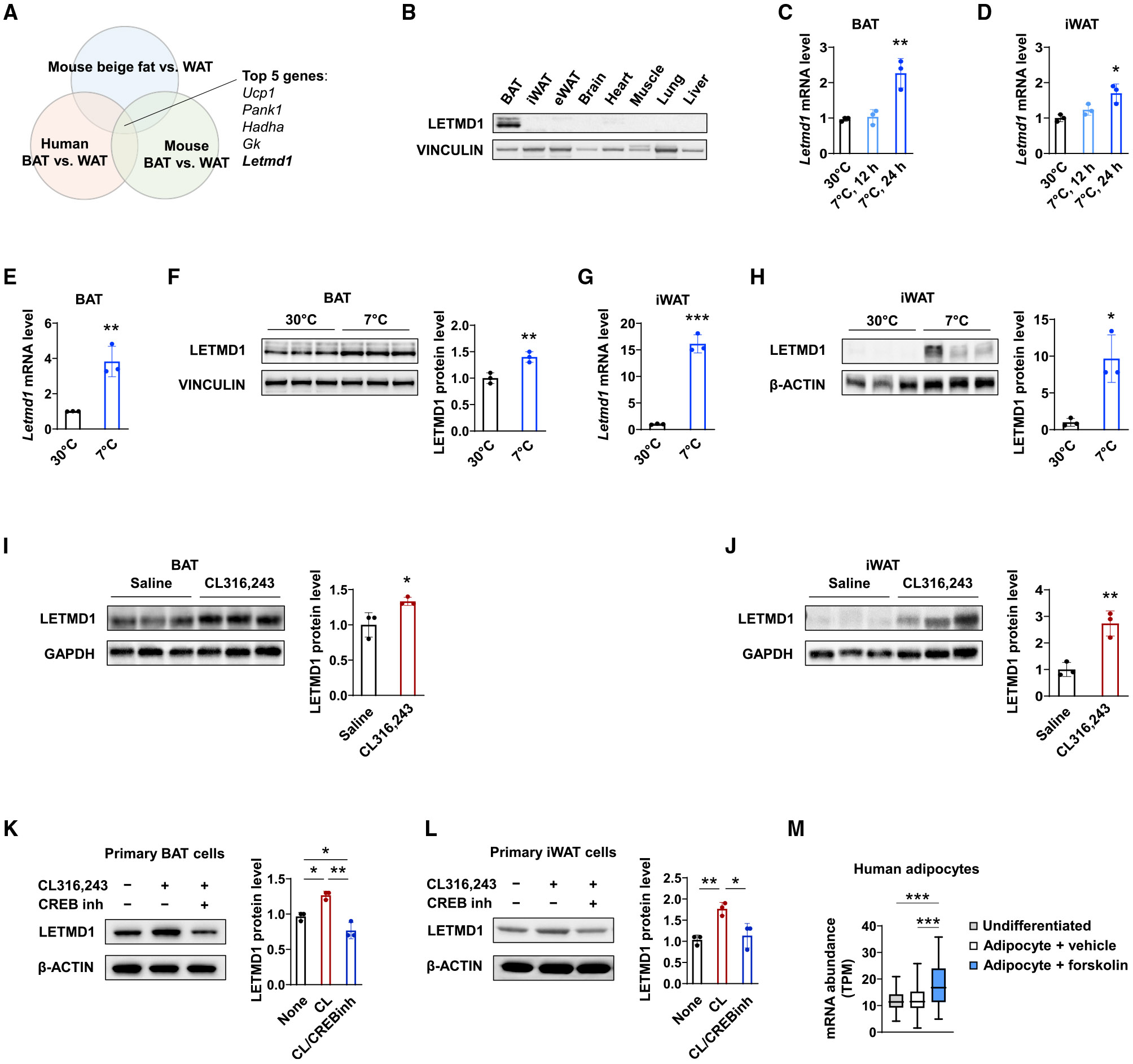
Letmd1 expression is highly enriched in brown fat and induced during cold exposure and adrenergic stimulation (A) Venn diagram of genes enriched in mouse BAT versus WAT, human BAT versus WAT, and mouse beige fat versus WAT. Top 5 genes based on fold change in mouse beige fat are indicated. (B) LETMD1 protein levels in various tissues from 3-month-old male mice with vinculin as a loading control. (C and D) The Letmd1 mRNA levels in BAT (C) and iWAT (D) from 8-week-old male mice after acute cold exposure. n = 3 for each group. (E and F) The Letmd1 mRNA levels (E) and protein levels (F) in BAT from male mice after 3 weeks of chronic cold exposure (7°C) starting at 12 weeks of age. n = 3 for each group. (G and H) The Letmd1 mRNA levels (G) and protein levels (H) in iWAT from male mice exposed to cold (7°C) for 3 weeks starting at 12 weeks of age. n = 3 for each group. (I and J) LETMD1 protein levels in BAT (I) and iWAT (J) from 10-week-old male mice administered saline or CL316,243 for 7 days. n = 3 for each group. n = 3 for each group. (K and L) BAT stromal vascular fraction (SVF) cells (K) and iWAT SVF cells (L) treated with 10 μM CL316,243 and 1 μM CREB inhibitor (666-15; Sigma) for 48 h. n = 3 for each group. (M) Expression of Letmd1 mRNA in cultured human adipocytes, comparing undifferentiated, vehicle-treated, and forskolin-treated adipocytes (10 μM for 3 days). TPM, transcripts per million. From publicly available RNA sequencing data (GEO: GSE134570). Bar graphs are presented as the mean ± SD; p values were determined by two-tailed Student’s t test. See also Figure S1 and Table S2.

We reasoned that genes that enhance adipocyte thermogenesis are likely to be enriched in expression in thermogenic fat tissues such as brown and beige fat compared to non-thermogenic fat tissues. To identify possible candidates, we examined a published list of 119 genes common to both human and mouse brown fat, termed the BATLAS, which was generated by combining the transcriptome data from mouse brown and white adipocyte populations purified by fluorescence-activated cell sorting (FACS) and from biopsy samples of human deep neck BAT and subcutaneous WATs (Perdikari et al., 2018). Requiring an overlap with the human data increased the chance of relevance to human physiology. To select for genes operative in both brown and beige fat thermogenesis, we cross-referenced this BATLAS list to our transcriptome data on inguinal WAT (iWAT) from mice exposed to cold temperatures for a prolonged period (7°C for 3 weeks) compared to controls housed at thermoneutrality (30°C). Based on the fold change of gene expression in the cold, the top-ranking gene on the overlapping list was Ucp1 followed by three enzymes of lipid metabolism (Figure 1A; Table S2). The next gene on the list was Letmd1, a mitochondrial outer membrane protein that has been implicated in tumorigenesis (Cho et al., 2007; Ha et al., 2010). Letmd1 has sequence homology to Letm1, a mammalian ortholog of the putative *Saccharomyces cerevisiae* mitochondrial K^+^/H^+^ exchanger MDM38, and was reported to partially rescue the exchanger activity in mutant *S. cerevisiae* (Zotova et al., 2010). We decided to further analyze Letmd1 because it had features suggestive of a regulatory role and had not been studied in the context of thermogenesis.

According to publicly available mouse tissue gene expression profiles, the Letmd1 mRNA is detected at the highest level in BAT (Figure S1A). Its mRNA levels are relatively low in other mouse and human tissues examined (Figures S1A and S1B). We confirmed by immunoblotting that the Letmd1 protein is expressed highly in BAT, whereas its levels in inguinal WAT and epididymal WAT are very low (Figure 1B). The Letmd1 mRNA levels were induced in BAT and iWAT within 24 h upon cold exposure compared to those at thermoneutrality (30°C) (Figures 1C and 1D). Following chronic cold exposure, the Letmd1 mRNA and protein levels were induced in BAT and iWAT (Figures 1E-1H), and the increases in iWAT appeared more striking because of the very low basal levels.

We examined if the Letmd1 levels could also be induced by treatment with CL316,243, a β3-adrenergic agonist that elicits many of the physiological responses to cold exposure in adipose tissue, including lipolysis and thermogenesis (Liu et al., 1998). The induction of Letmd1 with CL316,243 treatment was observed in both BAT and iWAT tissues *in vivo* (Figures 1I and 1J), as well as in cultured primary BAT cells and iWAT cells differentiated from the corresponding stromal vascular cells (Figures 1K and 1L). Notably, the CL316,243-mediated increase in Letmd1 levels was abrogated by simultaneous treatment with 666-15, a selective cyclic AMP (cAMP) response element binding protein (CREB) inhibitor, indicating that the induction requires cAMP-dependent gene transcription (Figures 1K and 1L). Public transcriptome data from cultured human adipocytes (Min et al., 2019) show that Letmd1 expression is increased with stimulation of cAMP signaling by forskolin (Figure 1M), suggesting conservation between human and mouse in this respect. Together, these data indicate that the Letmd1 expression correlates closely with the activation of thermogenesis in adipocytes and its induction likely involves the β-adrenergic-receptor-mediated cAMP signaling pathway.

### Letmd1-deficient mice exhibit defective thermogenesis in BAT

**Figure 2.**
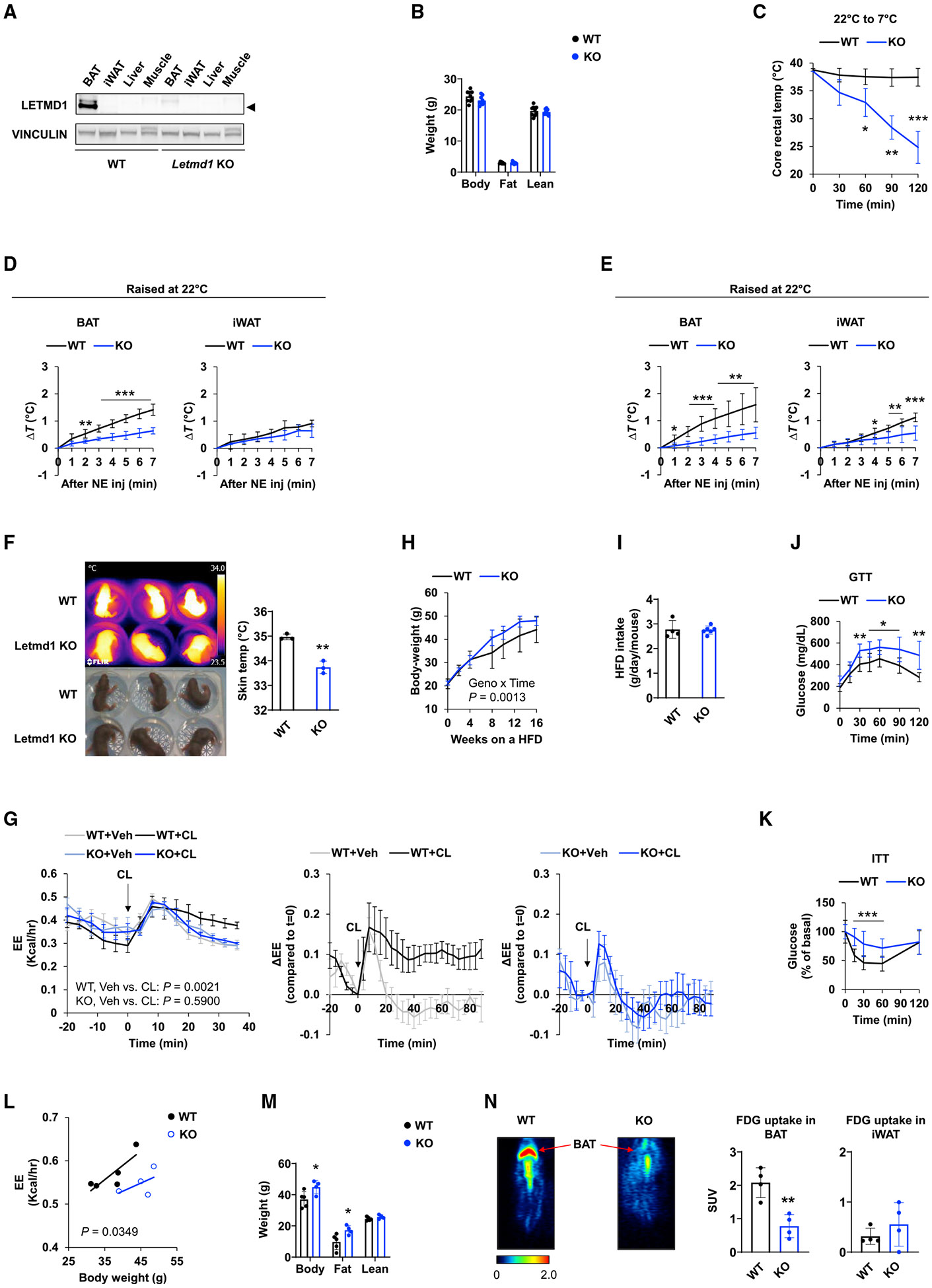
Letmd1 KO mice have severely impaired brown fat thermogenesis and are prone to diet-induced obesity and abnormal glucose metabolism (A) LETMD1 protein levels in tissues from 6-week-old female Letmd1 KO and WT control mice. (B) Dual-energy X-ray absorptiometry (DEXA) analysis of body composition in 13-week-old male Letmd1 KO versus WT mice at 22°C. n = 9 for both groups. (C) Core body temperature in 8-week-old male Letmd1 KO and WT mice during acute cold exposure. n = 4 for both groups. (D and E) Changes in tissue temperature following norepinephrine (NE) treatment. Seven-week-old male Letmd1 KO and WT mice were kept at 22°C (D) or adapted at 15°C for 3 days then housed at 7°C for 3 weeks (E). The temperature measurements were performed at 10 weeks. n = 6 for each group. (F) Infrared thermal images (top) and photographic images (bottom) of P4 newborns. Bar graph indicates average surface temperature from infrared images. n = 3 for both groups. (G) Energy expenditure of 20-week-old male Letmd1 KO and WT mice after administration of CL316,243 (CL) or saline (Veh). WT + Veh, n = 6; WT + CL, n = 6; KO + Veh, n = 7; KO + CL, n = 7. Data are mean ± SEM. (H) Body weight of male WT (n = 8) and KO mice (n = 5) fed a high-fat diet (HFD) starting at 6 weeks of age. (I) Daily food intake during HFD feeding. WT, n = 4; KO, n = 6. (J) Glucose tolerance test in 22-week-old male WT (n = 7) and KO mice (n = 5) on a HFD. (K) Insulin tolerance test in 20-week-old male WT and KO mice on a HFD. n = 8 for both groups. (L) Energy expenditure of 22-week-old male WT (n = 5) and Letmd1 KO mice (n = 4) on a HFD. (M) Body composition analysis of 22-week-old male WT (n = 5) and Letmd1 KO mice (n = 4) on a HFD. (N) [^18^F] FDG-PET images of 22-week-old male WT and Letmd1 KO mice on a HFD and [^18^F] FDG standardized uptake value (SUV) in BAT and iWAT. n = 4 each. Graphs are presented as mean ± SD unless otherwise noted. The p values were determined by two-tailed Student’s t test except for data in (G) and (H), for which we used two-way analysis of variance (ANOVA) followed by Fisher’s least significant difference (LSD) test, and in (L), for which we used analysis of covariance (ANCOVA). See also Figure S2 and ^S3^.

To evaluate a possible functional role for Letmd1 in thermogenesis, we generated Letmd1 knockout (KO) mice by CRISPR-Cas9 gene editing in mouse zygotes that created a deletion of exon 4. We confirmed the deletion of Letmd1 in tissues including BAT (Figure 2A). Relative to the wild-type (WT) mice, the Letmd1 KO mice (13 weeks) did not differ significantly in body weight when housed at 22°C (Figure 2B). When acutely exposed to cold temperatures (7°C), the KO mice (8 weeks) had severe difficulty maintaining their core body temperature (Figure 2C), suggesting a defect in BAT-mediated thermogenesis. To assess local heat generation, we directly monitored real-time changes in tissue temperature following norepinephrine (NE) treatment using thermocouple probes implanted in BAT and iWAT (Figures 2D and 2E) of 10-week-old WT and KO mice. We observed that the NE-induced temperature increase was blunted in the KO BAT, indicating an impaired ability to produce heat in response to adrenergic stimulation (Figure 2D). Among animals housed at cold (7°C) for 3 weeks, which was preceded by 3 days at 15°C to allow for incremental adaptation, the KO mice exhibited reduced temperatures in both BAT and iWAT, suggesting that in the KO mice, beige fat thermogenesis is defective along with BAT thermogenesis (Figure 2E). We also found that newborn (postnatal day 4 [P4]) KO mice had lower surface temperatures when imaged with a thermal camera, consistent with a thermogenic defect being already present at birth (Figure 2F). Male mice were used for these studies, but female mice had comparable levels of Letmd1 protein expression in BAT and were also cold intolerant (Figures S2A and S2B), suggesting similarities in the physiological role of Letmd1 regardless of the sex.

Differences in the baseline average 24-h energy expenditure or energy intake between the KO and WT animals did not reach statistical significance at 22°C (Figures S3A and S3B). High-definition indirect calorimetry, however, revealed that Letmd1 regulates β3-adrenoreceptor-dependent thermogenesis *in vivo* (Figure 2G). Following intraperitoneal injection of CL316,243 (1 mg/kg), WT mice (20 weeks) responded with an immediate increase in whole-body energy expenditure that was sustained over time, but WT mice injected with vehicle (saline) or Letmd1 KO mice injected with vehicle or CL316,243 failed to mount a similarly sustained energy expenditure increase. We also assessed a role for Letmd1 in the context of diet-induced obesity. Mice lacking Letmd1 showed accelerated weight gain compared to WT when fed a high fat diet (HFD) at room temperature (RT; 22°C), starting at 6 weeks of age (Figure 2H). There were no differences in food intake between WT and KO (Figure 2I). In addition to a higher body weight, the KO mice displayed deteriorated glucose tolerance and insulin sensitivity (Figures 2J and 2K). Indirect calorimetry showed a lower baseline energy expenditure in the HFD-fed KO mice compared to WT mice (20 weeks) (Figure 2L). Body composition analysis demonstrated increased fat mass but similar lean mass in the KO animals (Figure 2M). On [^18^F] fluorodeoxyglucose (FDG)-positron emission tomography (PET) imaging, which is commonly used to detect thermogenic adipose tissue in humans and mice (Chen et al., 2016; Cypess et al., 2013, 2015; Wang et al., 2012; Zhang et al., 2018), the HFD-fed KO mice (22 weeks) had a much lower [^18^F]-FDG uptake in BAT than WT mice, whereas the uptake in iWAT was not different (Figure 2N). This finding provides visual evidence of impaired thermogenesis in BAT in the HFD-fed KO mice. Collectively, these data reveal an *in vivo* function for Letmd1 in controlling whole-body energy balance, with implications for systemic metabolic health.

### Loss of Letmd1 impairs brown fat development and thermogenic gene expression

**Figure 3.**
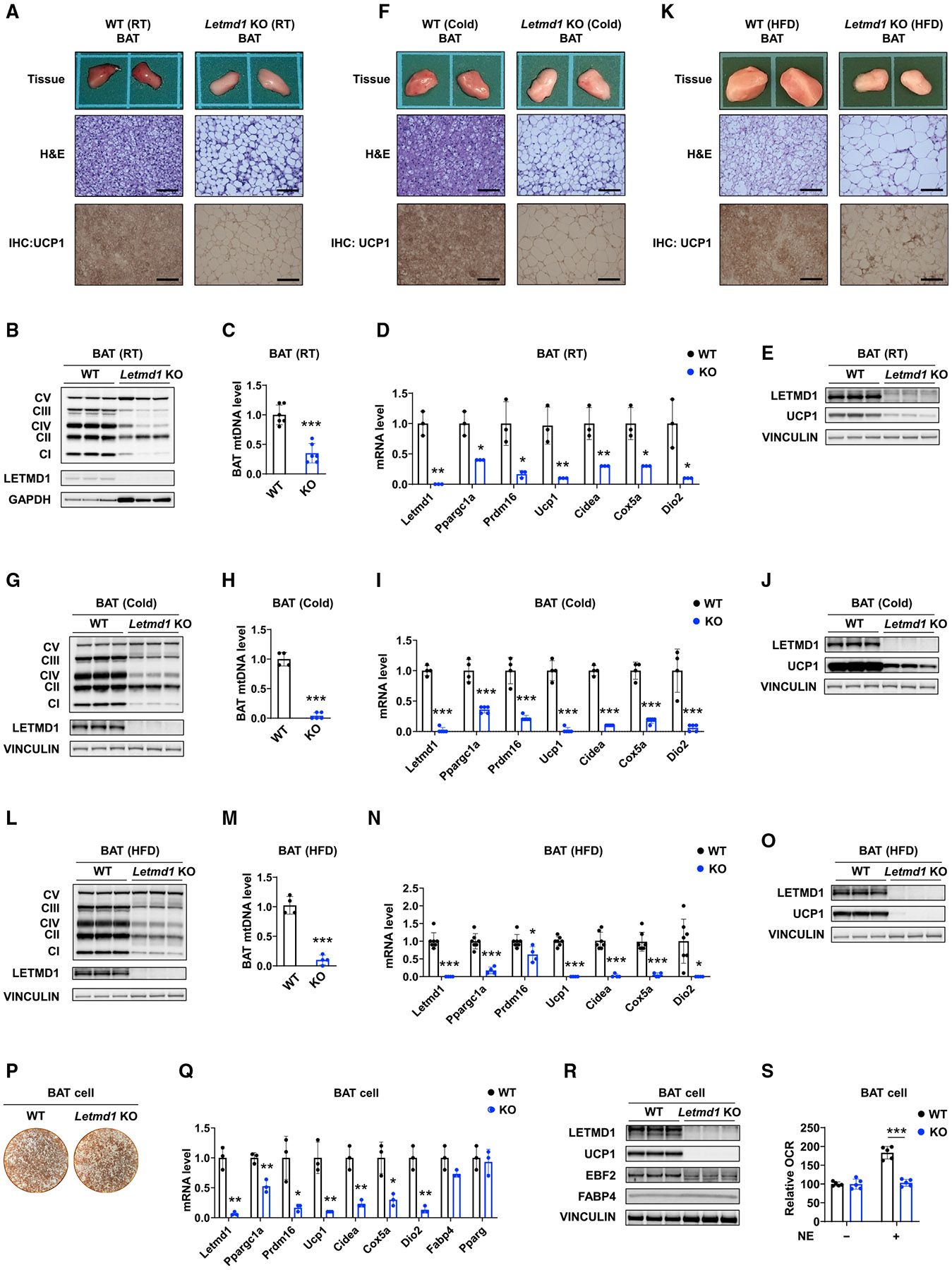
Letmd1 is required for normal BAT formation and thermogenic gene expression (A) Gross appearance (top), histological appearance (203) after hematoxylin-eosin (H&E) staining (middle), and IHC for UCP1 (bottom) of BAT from 10-week-old male WT and Letmd1 KO mice. Scale bar, 100 μm. (B) Protein levels of mitochondrial complex components in the BAT from 10-week-old male WT and KO mice. n = 3 for both groups. (C) Mitochondrial DNA (mtDNA) levels in BAT from 10-week-old male WT and KO mice. The mtDNA levels were determined by measuring relative levels of MT-Nd1 and Hk2. n = 6 for both groups. (D) mRNA expression of thermogenic genes in BAT from 10-week-old male WT and KO mice raised at RT, analyzed by qPCR. n = 3 for both groups. (E) Protein expression of UCP1 in BAT from 10-week-old WT and KO mice raised at RT. (F–J) Tissue images (F), protein levels of mitochondrial complex (G), mtDNA levels (H), thermogenic gene expression (I), and UCP1 protein expression (J) in BAT from male WT and KO mice housed at 7°C for 3 weeks starting at 7 weeks of age. The Letmd1 and vinculin blots from (G) and (J) are the same blots. For (F), scale bar, 100 μm. For (H) and (I), WT, n = 4; KO, n = 5. (K–O) Tissue images (K), protein levels of mitochondrial complex (L), mtDNA levels (M), thermogenic gene expression (N), and UCP1 protein expression (O) in BAT from 22-week-old male WT and KO mice on a HFD. The Letmd1 and vinculin blots from (L) and (O) are the same blots. For (K), scale bar, 100 μm. For (M), n = 4 each. For (N), WT, n = 7; KO, n = 4. (P) Differentiated BAT cells from WT and KO mice stained with oil red O. (Q) qPCR analysis of mRNA expression of thermogenic genes and adipogenic genes in BAT cells isolated from WT and KO mice. n = 3 for each group. (R) Expression of proteins involved in thermogenesis and lipogenesis in BAT cells isolated from WT and KO mice. (S) Relative oxygen consumption rate (OCR) in WT and KO BAT cells treated with 10 μM NE or vehicle. n = 5 for each group. Bar graphs are presented as the mean ± SD. The p values were determined by two-tailed Student’s t test. See also Figure S4.

To understand the mechanisms underlying the defective regulation of thermogenesis and energy balance in the Letmd1 KO mice, we first examined BAT in 10-week-old animals and found that BAT from KO mice was much lighter in color than that in the WT (Figure 3A). H&E staining revealed large unilocular lipid droplets resembling WAT rather than small multilocular lipid droplets typically seen in thermogenic fat tissue (Figure 3A). Because the characteristic brown color in BAT comes from iron-rich mitochondria, this finding suggested a decrease in mitochondrial abundance. Indeed, we found reduced levels of representative subunits from the 5 mitochondrial electron transport complexes as well as decreased mitochondrial DNA levels in BAT from Letmd1 KO (Figure 3B, C). We also confirmed a marked reduction in KO BAT of the expression of several key regulators of BAT development and thermogenesis, such as Ucp1, PGC1α, Prdm16, Cidea, Cox5a, and Dio2, by using qPCR (Figure 3D). Decreased levels of the UCP1 protein expression were observed by immunohistochemistry (IHC) and western analysis (Figures 3A and 3E). Even when mice were chronically housed at 7°C, BAT from the Letmd1 KO mice still had the morphological appearance of WAT and low mitochondrial electron transport complex protein and mitochondrial DNA levels, as well as reduced thermogenic gene expression and UCP1 protein expression (Figures 3F-3J). This result offers a contrast with other mouse models such as the adipose-specific Ebf2 KO mice, which are cold sensitive but gradually recover normal BAT morphology and function during chronic cold exposure (Angueira et al., 2020). BAT from the KO mice fed a HFD also had larger lipid droplets than BAT from similarly treated WT mice and was considerably more difficult to distinguish from the surrounding WAT (Figure 3K). Reduced levels of mitochondrial DNA and electron transport complex proteins, thermogenic gene expression, and UCP1 protein expression were again observed (Figures 3K-3O). We also examined liver tissues from the WT and KO mice. On a regular diet, there were no appreciable differences between WT and KO mice in liver weight, gross and microscopic morphology, or expression of genes related to inflammation or lipid metabolism (Figures S4A, S4B, and S4C). The liver from HFD-fed Letmd1 KO mice, on the other hand, weighed more than their WT counterparts and contained some regions of steatosis without apparent changes in the inflammatory and lipid metabolism genes (Figures S4D, S4E, and S4F).

In differentiated brown adipocytes, the loss of Letmd1 did not affect lipid droplet accumulation as assessed by oil red O staining (Figure 3P). Consistent with this finding, KO brown adipocytes had similar expression levels of general adipogenesis genes such as Fabp4 and Pparg compared to WT adipocytes (Figure 3Q). However, thermogenesis-related genes were reduced in KO brown adipocytes (Figure 3Q). Immunoblotting confirmed lower levels of thermogenic markers such as UCP1 and EBF2 in KO cells, whereas the adipogenic marker FABP4 was preserved (Figure 3R). Deletion of Letmd1 also abolished the NE-mediated increases in oxygen consumption in brown adipocytes (Figure 3S). These data suggest that the loss of Letmd1 profoundly impairs BAT formation and function in part because Letmd1 regulates the expression of key BAT thermogenesis genes in a cell-autonomous fashion.

**Figure 4.**
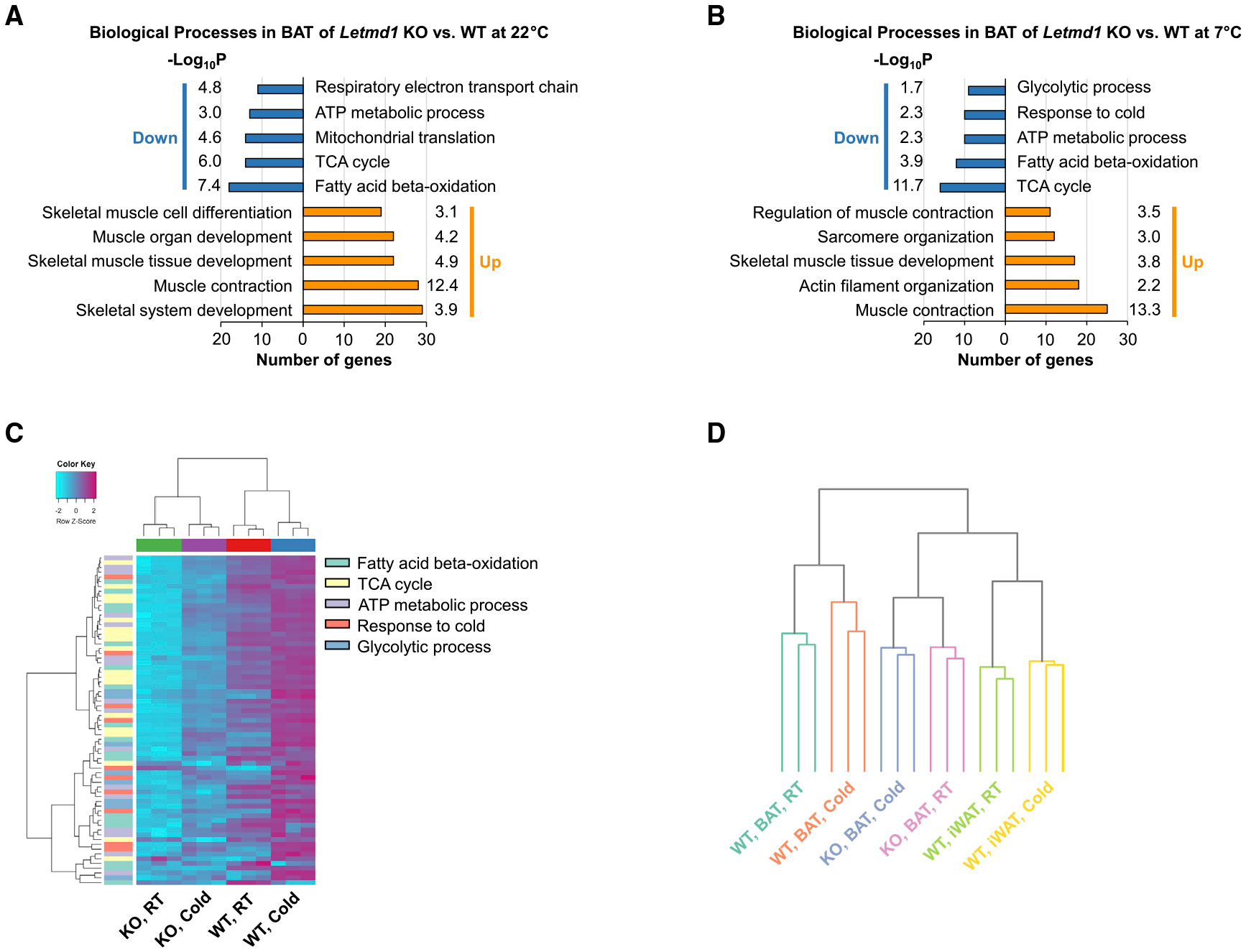
Loss of Letmd1 results in broad-ranging deficiencies in the transcriptional program associated with cold adaptation (A and B) Enriched biological processes in BAT from male Letmd1 KO versus WT control mice raised at 22°C or 7°C for 3 weeks starting at 7 weeks of age. (C) Hierarchical clustering for genes included in downregulated biological process terms in (A) and (B). (D) Hierarchical clustering of whole-transcriptome data from WT BAT, Letmd1 KO BAT, and WT iWAT mice exposed to 7°C (cold) or 22°C (RT) for 3 weeks starting at 7 weeks of age.

We next performed RNA sequencing on BAT from the Letmd1 KO and WT mice housed at 22°C or at 7°C to assess general alterations in the transcriptome. We identified 1,907 upregulated genes and 1,405 downregulated genes in KO compared to those of the WT at 22°C, and 1,234 upregulated genes and 784 downregulated genes at 7°C (fold change, >2; false discovery rate (FDR)-adjusted p < 0.05). Pathway analysis showed that both at 22°C and at 7°C, genes linked to the tricarboxylic acid (TCA) cycle, electron transport, and fatty acid oxidation were reduced in the KO (Figures 4A and 4B). On the other hand, genes involved in muscle differentiation and contraction were induced in the KO, suggesting that Letmd1 suppresses muscle genes (Figures 4A and 4B). Notably, even under cold conditions, the expression levels of genes related to mitochondrial function and thermogenesis in KO mice did not reach the levels seen in WT at RT, indicating that regulation of the cold-induced transcriptome is severely compromised by the loss of Letmd1 (Figure 4C). Hierarchical clustering analysis of the transcriptome profiles showed that KO BAT is closer to WT iWAT than to WT BAT (Figure 4D). Thus, the RNA sequencing data demonstrate that the deletion of Letmd1 profoundly impairs the brown adipocyte transcriptional program on a broad scale, particularly those associated with brown adipocyte cell fate determination, mitochondrial metabolism, and cold adaptation.

### Letmd1 interacts with Brg1 in the nucleus to regulate thermogenesis

**Figure 5.**
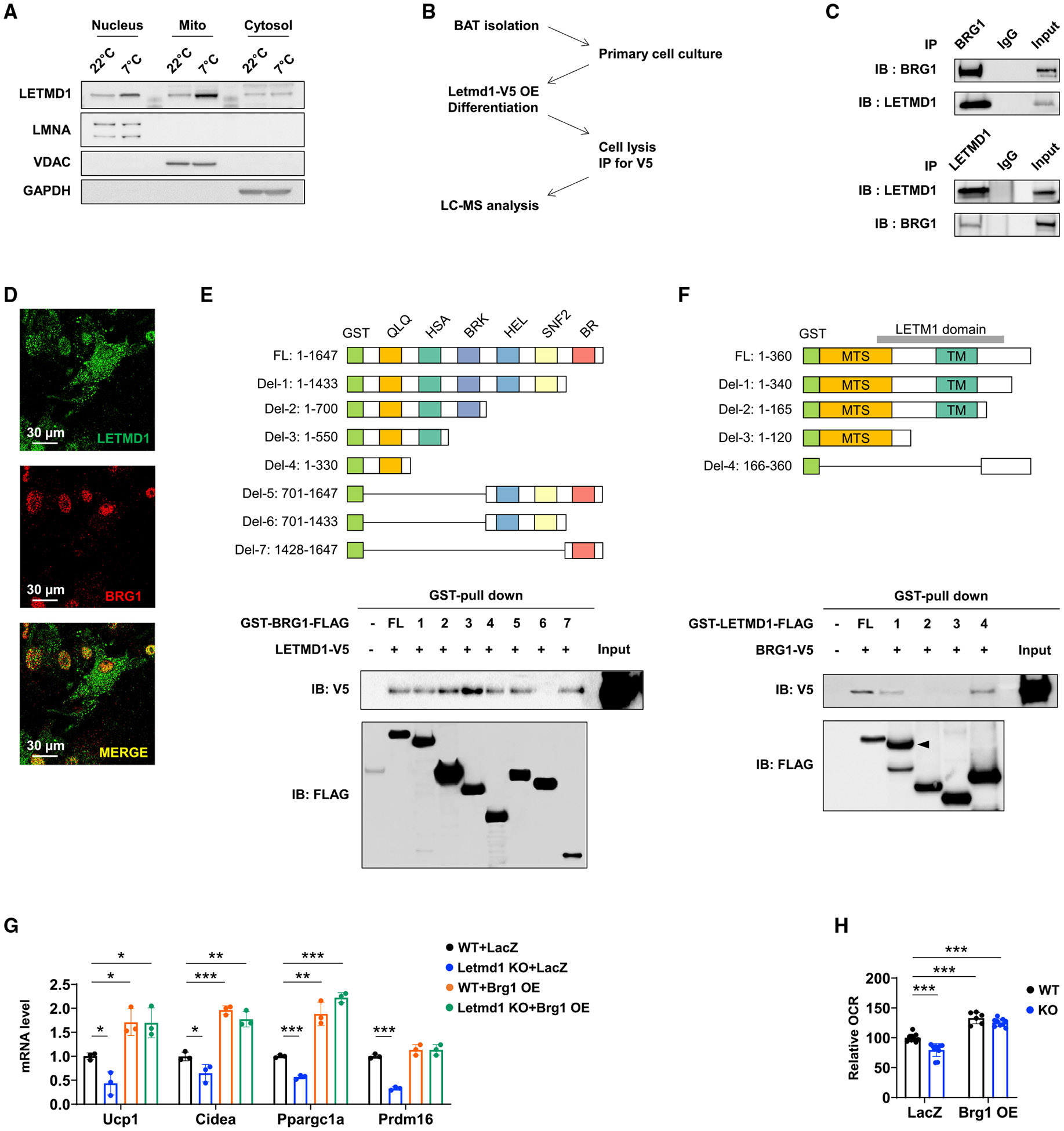
Letmd1 interacts with Brg1 in the nucleus to regulate thermogenic gene expression and respiration (A) Subcellular localization of Letmd1 in BAT of male mice raised at 22°C or 7°C for 2 weeks starting at 10 weeks. (B) Immunoprecipitation (IP) and tandem mass spectrometry (MS)-based strategy for identification of Letmd1-interacting proteins. (C) Immunoblot after IP for BRG1 (top) and LETMD1 (bottom) in BAT cells. (D) Immunofluorescence images for LETMD1 and BRG1 in BAT cells. (E and F) Schematic diagrams of GST-tagged BRG1 (E) and GST-tagged LETMD1 (F) are shown. Immunoblot images of GST pull-down for each protein is shown below. FL, full length; HEL, helicase domain; SNF2, Snf2-ATP coupling; BR, bromodomain; MTS, mitochondrial targeting sequence; TM, transmembrane domain. (G and H) Brg1 overexpression (OE) compensates for Letmd1 loss. BAT SVF cells isolated from WT and Letmd1 KO mice were transduced with Brg1 virus or with LacZ control virus, differentiated, and treated with 10 μM NE for 1 h. mRNA expression (G) and OCR (H) were assayed under the indicated conditions. For (G), n = 3 for each condition. For (H), WT + LacZ, n = 11; KO + LacZ, n = 11; WT + Brg1 OE, n = 6; KO + Brg1 OE, n = 9. Bar graphs are presented as the mean ± SD. The p values were determined by two-tailed Student’s t test. See also Table S3.

We were struck by the fact that a mitochondrial protein could have such dominant effects on gene expression and decided to examine its subcellular localization by fractionation. The Letmd1 protein was present in the nuclear fraction as well as the mitochondrial fraction, and its levels increased in abundance in both fractions following cold exposure (7°C) (Figure 5A). This result raised the possibility that Letmd1 could control gene expression by directly interacting with transcriptional regulators. To identify interaction partners of Letmd1, we overexpressed Letmd1 in brown adipocytes and performed immunoprecipitation followed by shotgun mass spectrometry (Figure 5B). Among the transcription or epigenetics-related nuclear proteins identified by this strategy (Table S3), the chromatin remodeling protein Brg1 (also known as Smarca4) attracted our attention because it is a component of the ATP-dependent chromatin remodeling complex BAF (mammalian SWI/SNF complex) reported to partner with the brown fat differentiation transcription factor Ebf2 and the histone demethylase JMJD1A, of which both have been linked to regulation of thermogenesis (Abe et al., 2015; Shapira et al., 2017). Brg1 is the catalytic ATPase in the BAF complex and controls transcriptional activation and repression of specific genes in a context-dependent fashion by chromatin remodeling. We validated the physical interaction between Letmd1 and Brg1 by immunoprecipitationwith Brg1 as bait and also with Letmd1 as bait (Figure 5C). Co-localization of Letmd1 and Brg1 in the nucleus was demonstrated by confocal immunofluorescence microscopy (Figure 5D). Although Letmd1 is also detected in mitochondria, the Brg1 protein is restricted to the nucleus. We mapped the interaction domains of Letmd1 and Brg1 by glutathione S-transferase (GST) fusion protein pull-down assays (Figures 5E and 5F). The C-terminal region of Letmd1 is involved in the interaction with Brg1, whereas either the N-terminal or the C-terminal domains within the Brg1 protein is sufficient for binding.

Brg1 has previously been shown to be required for the expression of Ucp1 and other brown-fat-specific genes but not for general adipogenesis genes (Shapira et al., 2017). When we ectopically expressed Brg1 in the KO brown adipocytes, thermogenic gene expression and NE-mediated respiration were rescued (Figures 5G and 5H). This result identifies Brg1 as a downstream effector of Letmd1-dependent thermogenic gene regulation.

**Figure 6.**
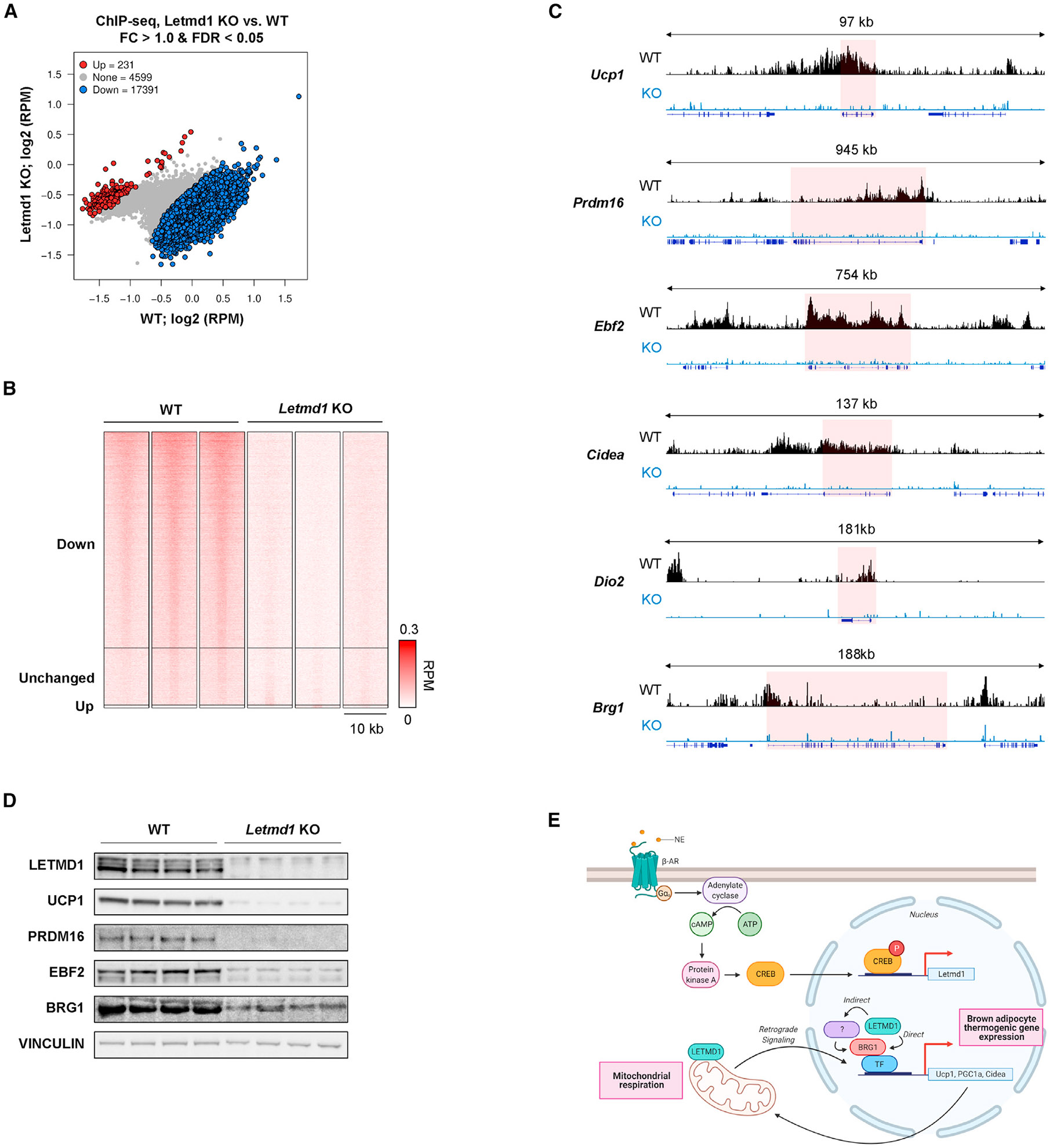
Chromatin binding of BRG1 requires Letmd1 (A) Differential binding analysis for chromatin IP sequencing (ChIP-seq) data from WT versus Letmd1 KO BAT (FDR, <0.05). (B) Heatmap visualization for (A). (C) Genome browser tracks for ChIP-seq data around key thermogenic genes. Red box indicates locus for the indicated gene. (D) Protein expression in BAT from 10-week-old male WT and KO mice. (E) A suggested model of thermogenic regulation by Letmd1 in brown adipocytes. Created with BioRender.com.

Because of the apparent importance of the nuclear protein Brg1, a component of the BAF chromatin remodeling complex that ultimately controls transcription factor access to gene promoters, we hypothesized that the loss of Letmd1 affects the pattern of Brg1 binding to individual gene promoters. We conducted a chromatin immunoprecipitation (ChIP) sequencing analysis of BAT from the Letmd1 KO and WT mice by using a Brg1 antibody. Brg1 binding to chromatin was globally diminished in the KO BAT, as seen in a differential analysis and heatmap visualization (Figures 6A and 6B). Decreased recruitment of Brg1 was evident at many BAT development and thermogenesis genes such as Ucp1, Prdm16, Ebf2, Cidea, and Dio2, as well as Brg1 itself (Figure 6C). These data are consistent with gene expression analysis and western analysis of BAT from WT and KO mice (Figure 3D; Figure 6D) that show markedly reduced levels of key genes controlling BAT development and function.

## DISCUSSION

In this study, we have identified a role for Letmd1 as a key regulator of BAT formation and thermogenic function. The expression of Letmd1 is highly selective for thermogenic adipose tissues (Figures 1B and S1A) and upregulated in response to cold stress or β-adrenergic stimulation (Figures 1C-1M). The loss of Letmd1 in mice results in marked reduction in the levels of key BAT development and thermogenesis genes (Figures 4A-4C). Animals that lack Letmd1 have abnormal BAT morphology (Figures 3A, 3F, and 3K) and dramatically reduced ability to produce heat (Figures 2C-2G), as well as a predisposition to diet-induced obesity and impaired glucose disposal (Figures 2H-2K).

We initially focused our attention on Letmd1 because of its strong enrichment in BAT versus WAT in both human and mouse transcriptome datasets and also in beige fat versus WAT, implying a close connection with thermogenic capacity that is preserved across species (Figure 1A). Its sequence homology to Letm1, a mammalian ortholog of a putative yeast mitochondrial K^+^/H^+^ exchanger (Zotova et al., 2010), suggested a potential for a regulatory role. Letmd1 has also been implicated in the regulation of p53 and tumorigenesis (Cho et al., 2007; Ha et al., 2010). Although Letmd1 was originally identified as a mitochondrial outer membrane protein, we have found that it is present in both the nucleus and mitochondria (Figure 5A), which makes it somewhat atypical among thermogenesis regulators. Gene expression abnormalities due to a disruption of its nuclear function can explain the defective BAT development, reduced mitochondrial biogenesis, and impaired thermogenesis responses seen in Letmd1 KO mice. On the other hand, Letmd1 can still have important functions in mitochondria that can affect thermogenesis and may or may not overlap with its nuclear function (Figure 6E). A number of mitochondrial proteins have been described to translocate to the nucleus, possibly representing a mechanism by which mitochondria signals to the nucleus to alter transcriptional responses (Monaghan and Whitmarsh, 2015). For example, oxidative stress triggers the transcription factor NRF2 to translocate from mitochondria to the nucleus (Lo and Hannink, 2008; Paek et al., 2012; Yamamoto et al., 2018), whereas p53 translocates from the nucleus to mitochondria in response to various apoptosis-inducing stress signals (Holley and St Clair, 2009; Lindenboim et al., 2011). Dual localization of Letmd1 in the nucleus as well as mitochondria likely allows for dynamic interactions with distinct sets of partner proteins in a compartment-specific fashion. It will be worthwhile to identify such compartment-specific interacting proteins of Letmd1 to fully understand its cellular functions.

Our data indicate that Letmd1 expression is not only enriched in thermogenic tissues but also controlled by physiological activators of BAT thermogenesis such as cold or adrenergic signaling (Figures 1C-1M). β3-Adrenergic signaling is widely considered to be a dominant pathway governing fat thermogenesis in the context of cold exposure. Elevated levels of cAMP that result from β3-receptor stimulation promote activation of protein kinase A (PKA), which in turn targets downstream molecules such as p38 mitogen-activated protein kinase (MAPK), CREB, and hormone sensitive lipase (HSL) (Mayr and Montminy, 2001; Tabuchi and Sul, 2021). The induction of Lemd1 in cultured mouse adipocytes by the β3-adrenergic agonist CL315,243 is blocked by a CREB inhibitor (Figures 1K and 1L), and the Letmd1 level in human adipocytes is increased by forskolin, a stimulator of cAMP signaling (Figure 1H), pointing to a key role for the cAMP-PKA signaling pathway in regulating Letmd1 gene expression. Furthermore, the loss of Letmd1 essentially eliminates β3-adrenoreceptor-dependent energy expenditure *in vivo* (Figure 2G), indicating a critical functional role for Letmd1 in mediating the physiological effects of this signaling pathway. These observations strongly suggest that Letmd1 links adrenergic signaling to thermogenesis (Figure 6E). As noted earlier, some thermogenesis-related proteins such as Prdm16 are not acutely regulated by cold or adrenergic stimulation (Seale et al., 2007). Letmd1 mice exhibit apparent thermogenic defects at birth (Figure 2F), whereas Prdm16-deficient mice are born with fully functional BAT, although the thermogenic capacity declines gradually later in life (Harms et al., 2014).

Another difference lies in tissue expression patterns. BAT is the primary site of expression for Letmd1 (Figures 1B and S1A). In contrast, Prdm16 and Ebf2 exhibit more widespread tissue expression and regulate the development of vital tissues such as the brain, hematopoietic cells, and the cardiac conduction system (Aguilo et al., 2011; Bjork et al., 2010; Chuang et al., 2011; Kieslinger et al., 2010; Nam et al., 2020), which may explain why Prdm16 KO and Ebf2 KO are embryonic lethal. The Letmd1 KO mice are viable and grow normally (Figure 2B), and yet its BAT phenotype is more severe, resulting in defective BAT formation (Figures 3A, 3F, and 3K), impaired acute and chronic cold adaptation (Figures 2C and 4C), and increased susceptibility to diet-induced obesity (Figures 2H and 2I). The adipose-specific Ebf2 KO mice are sensitive to acute cold stress but have a normal chronic cold adaptation with normal-appearing BAT (Angueira et al., 2020), which is attributed to compensation by related Ebf proteins. They also have intact diet-induced thermogenesis on a HFD (Angueira et al., 2020). On the other hand, the Letmd1 KO mice tolerate a prolonged cold exposure only if acclimated gradually, and their BAT remains abnormal morphologically and functionally (Figures 3F-3J). Because of its tissue-selective expression, Letmd1 may be better suited as a potential therapeutic target. Indeed, a number of agents already exist that can increase energy expenditure such as adrenergic stimulants, thyroid hormone, or even the uncoupling agent 2,4-dinitrophenol (Grundlingh et al., 2011; Tseng et al., 2010). These agents all produce weight loss but also deleterious off-target effects, making them unsuitable for use as therapeutics. A higher degree of tissue selectivity can be advantageous in helping to minimize off-target effects and increase the therapeutic window.

It is also this tissue selectivity that potentially makes Letmd1 well poised to play a role in BAT cell fate determination and function. Efforts to identify brown-adipocyte-lineage-specific transcriptional regulators over the past decade have led to important molecules such as Prdm16 and Ebf2 (Rajakumari et al., 2013; Seale et al., 2007). Ebf2 has been proposed to act as a pioneer factor early in BAT differentiation and is capable of binding target genomic sites in a closed chromatin state and recruiting the chromatin remodeler Brg1 and its associated BAF complex, with other transcriptional regulators such as PPARγ, Prdm16, and PGC1α becoming involved later (Gulyaeva et al., 2019; Shapira et al., 2017). Letmd1 is not a transcriptional regulator in itself but it physically interacts with Brg1 (Figure 5C), and the loss of Letmd1 results in reduced levels of Brg1 and Ebf2 (Figure 6D). There are several possible ways that Letmd1 can control gene expression (Figure 6E). One possibility is that Letmd1 directly regulates Brg1 activity or its access to target genomic sites such as Ebf2, Prdm16, and Brg1 itself, and therefore, deletion of Letmd1 disrupts the BAT transcriptional program and lowers the expression levels of these key regulators. Through its interactions with Brg1, Letmd1 may confer a level of tissue selectivity in target gene transcription beyond what is provided by Ebf2 or the BAF complex. Because Brg1 overexpression can rescue the effects of Letmd1 loss (Figures 5G and 5H), however, it may be that the loss of Brg1 is key and direct binding between Brg1 and Letmd1 is not critical. A second possibility is that Letmd1 acts in conjunction with or by other regulators. For example, it may control the Brg1 or Ebf2 amount by modulating the activity of specific ubiquitin ligases or other modifying enzymes that target these proteins to alter stability. Brg1 has been reported to be targeted by the proteasome and is susceptible to degradation and removal from chromatin in the absence of scaffold proteins that are themselves regulated during tissue differentiation (Chen and Archer, 2005; Cullen et al., 2009; Sohn et al., 2007). Lastly, the loss of mitochondrial Letmd1 may activate retrograde signaling pathways to change the expression of Brg1 and other thermogenesis genes in the nucleus. These possibilities, which are not mutually exclusive, can account for the near-global reduction in the expression of Brg1 target genes and other BAT regulator genes observed in the KO BAT (Figures 4A and 4B). While this manuscript was in preparation, the results of a study by Song et al., (2020) were submitted to the preprint server bioRxiv, and the authors reported that deletion of Letmd1 causes alteration of mitochondrial calcium, defective mitochondrial fission, and impairment of thermogenesis in BAT. Using publicly available gene expression data (GEO: GSE66921), those authors also noted that Letmd1 is induced in human subcutaneous fat following weight loss surgery. There is substantial evidence that bariatric surgery is associated with increased BAT thermogenesis and WAT browning in humans and rodents (Dadson et al., 2018; Chen et al., 2018; Moncada et al., 2016), raising the possibility that Letmd1 may be involved in this process.

Letmd1 has a negligible level of expression in iWAT at baseline but is induced during chronic cold exposure (Figures 1G and 1H), pointing to a potential involvement in beige fat thermogenesis. Indeed, real-time tissue temperature recordings following NE injection suggest defective beige fat thermogenesis in the Letmd1 KO mice (Figure 2E). Because Letmd1 affects thermogenesis in both BAT and beige adipose tissue, modulation of Letmd1 may produce a greater effect on energy balance than interventions that target BAT alone, which could be an advantage. We have seen that the loss of Letmd1 appears to have a deleterious effect on both cold adaptation and diet-induced obesity (Figures 2C-2J). It is not always the case that these two forms of non-shivering thermogenesis are regulated in concert; the adipose-specific Ebf2 KO mice, for example, are more cold sensitive but do not gain any more weight than control mice on a HFD (Angueira et al., 2020). Although there is considerable evidence that BAT plays a key role in diet-induced thermogenesis (Feldmann et al., 2009; Rothwell and Stock, 1979; Saito et al., 2020), the contributions of other tissues and the precise mechanisms of dietary thermogenesis are still being clarified. Because mice experience mild cold stress at 22°C, definitively answering whether Letmd1 in BAT is necessary for diet-induced thermogenesis may involve the use of tissue-specific conditional KO mouse models housed at thermoneutrality to completely eliminate cold stress. What is unquestionable so far is that Letmd1 has a wide-ranging effect on systemic metabolism and that the loss of Letmd1 function results in prominent metabolic dysfunction encompassing obesity, glucose intolerance, and insulin resistance (Figures 2H-2K). Conversely, given that Letmd1 is further induced when thermogenesis is activated, it is conceivable that increasing the Letmd1 levels in adipose tissue may confer metabolic benefits such as resistance to weight gain and improved glucose homeostasis.

In conclusion, our results from studying the Letmd1-deficient mice strongly implicate Letmd1 in the regulation of thermogenic adipose tissue formation and function. Letmd1 appears to be a critical component of the pathways that control thermogenic fat cell fate and adaptive thermogenesis, and many details remain to be uncovered about the mechanisms by which Letmd1 alters the nuclear gene expression program and mitochondrial function. Given the extensive presence of thermogenic fat in humans and the potential of harnessing these remarkable cells as a defense against obesity and associated metabolic disorders, a better understanding of these mechanisms will be important for the development of therapeutic strategies that target energy balance.

### Limitations of the study

Tissue-specific gene targeting will be useful to definitively exclude contributions from other tissues to the phenotypes observed in the Letmd1 KO mice. We have examined a role for Letmd1 in modulating nuclear gene expression, but further studies are needed to fully delineate Letmd1-mediated regulatory circuits operative in mitochondria and the nucleus.

## STAR★METHODS

### RESOURCE AVAILABILITY

#### Lead contact

Further information and requests for resources and reagents should be directed to and will be fulfilled by the Lead Contact, John C. Yoon (jcyoon@ucdavis.edu).

#### Materials availability

Plasmids and mouse lines are available from the Lead Contact upon request.

#### Data and code availability

- RNA-sequencing and ChIP-sequencing data have been deposited at NCBI GEO and will be publicly available. The accession numbers are listed in the Key resources table.
- This paper does not report original code.
- Any additional information required to reanalyze the data reported in this paper is available from the lead contact upon request.

### EXPERIMENTAL MODEL AND SUBJECT DETAILS

#### Mice

All animal experiments were performed according to the protocols approved by the UC Davis Institutional Animal Care and Use Committee (IACUC). The global Letmd1 knockout mice were generated by the UC Davis Mouse Biology Program via CRISPR/Cas9 gene editing. The mice were in C57BL/6J background. Male or female mice at postnatal day 4 (P4) or at 6 weeks of age or older were used in this study, as indicated in the figure legends. Mice were maintained on a standard chow (Teklad Global Rodent Diets) at the indicated temperature under a 12 h light/12 h dark cycle. For a high-fat diet (HFD) study, the knockout (KO) and the wild-type (WT) mice were housed at 22°C and fed a 60% HFD (Research Diets, D12492). Food intake and body weight were measured on a biweekly basis. All experiments were conducted with littermate controls.

#### Primary cell isolation and culture

Brown adipose tissue (BAT) was dissected and digested with 2.4 unit/ml dispase II (Sigma D4693) and 1.5 unit/ml collagenase D (Sigma 11088882001) at 37°C for 50 minutes with agitation following established methods (Aune et al., 2013). The stromal vascular fraction (SVF) containing preadipocytes was separated from the mature adipocytes by centrifugation at 700 g for 10 min. SVF cells were then plated on coated dishes in culture medium containing DMEM (ThermoFisher, 10569044) with 10% fetal bovine serum (FBS) and differentiated by treating fully confluent cells with differentiation induction medium (DMEM with 10% FBS, 5 μg/ml insulin, 0.5 mM isobutylmethylxanthine (IBMX), 5 μM dexamethasone, 125 μM indomethacin, 1 nM 3,3′,5-triiodo-L-thyronine (T3) and 1 μM rosiglitazone) at day 0. After 2 days, the medium was changed to differentiation maintenance medium (DMEM containing 10% FBS, 5 μg/ml insulin, 1 nM T3 and 1 μM rosiglitazone). The medium was replenished every two days until fully differentiated.

### METHOD DETAILS

#### Real-time PCR

Total RNA was isolated by Trizol (ThermoFisher) followed by purification using RNA mini spin columns (Epoch Life Science). The cDNA was synthesized using SuperScript IV reverse transcriptase (ThermoFisher). qPCR was performed using SYBR Green (Bio-Rad) in a Bio-Rad CFX real-time PCR system. Primers used in this study are listed in Table S1.

#### Western blotting

Protein extracts were prepared using RIPA buffer supplemented with protease inhibitor (Roche). Proteins were loaded in 4%–12% or 3%–8% NuPAGE gels (ThermoFisher) and transferred to nitrocellulose membrane. Primary antibodies used in this study were anti-Letmd1 (LSBio; LS-C335200), anti-Brg1 (Bethyl; A300-813A-T or Cell Signaling; 49360S), anti-Ucp1 (R&D systems; MAB6158), anti-Prdm16 (R&D systems; AF6295), anti-Ebf2 (R&D systems; AF7006), anti-OXPHOS (MitoSciences; MS604), anti-lamin (Santa Cruz Biotechnology; sc-376248), anti-VDAC (Cell Signaling; 4661T), anti-FABP4 (Santa Cruz; sc-271529), anti-Flag (GenScript; A00187), anti-V5 (ThermoFisher; R96025), anti-β-actin (Santa Cruz; sc-47778), anti-Gapdh (Santa Cruz; sc-25778), and anti-vinculin (Sigma, V9131).

#### Cold challenge

For chronic cold exposure, mice were group-housed at either at 30°C, 22°C, or 7°C for 3 weeks with free access to food and water. For acute changes in the core body temperature in response to cold, mice were transferred to 7°C and singly housed with free access to food and water. Rectal core body temperature was monitored using a thermometer (Bioseb) every 30 min for 2 hours.

#### Treatment with the β3-adrenergic agonist CL316,243

CL316,243 or saline was intraperitoneally administered to mice at a dose of 1 mg/kg body weight for 7 consecutive days. On the day after the last injection, the mice were sacrificed.

#### Indirect calorimetry

Energy expenditure was evaluated by indirect calorimetry in WT and Letmd1 KO male mice (n = 9 each) fed a chow diet in the Comprehensive Lab Animal Monitoring System (CLAMS, Columbus Instruments). Animals were acclimated to the facility for 1 week, and to the CLAMS cages for 48 hours prior to initiation of calorimetry. Calorimetry data were collected for 48 hours at 22°C. For the HFD group, we evaluated WT and Letmd1 KO male mice (22 weeks of age, n = 5 and 4) on a 60% HFD and collected calorimetry data for 48 hours. In a separate study, the energy expenditure response to an IP injection of a β-adrenergic agonist (CL316,243, 1 mg/kg body weight) or vehicle was assessed by high resolution indirect calorimetry in male WT and KO mice fed a chow diet (20 weeks of age, n = 6 and 7). Measurements were made every 4 minutes starting 30 minutes before injection until 90 min post. The body composition was assessed by dual-energy X-ray absorptiometry (DEXA) under isoflurane anesthesia, using a Lunar PIXImus II Densitometer (GE Medical Systems) immediately after completion of the indirect calorimetry measurements.

#### Measurement of tissue temperature

To measure the local tissue temperature directly, thermocouple probes (Sable systems international, TC-2000) were implanted in BAT and iWAT of mice anesthetized with 1%–4% isoflurane. Temperature changes following intraperitoneal injection of norepinephrine (1 mg/kg) were recorded in real-time.

#### Infrared thermography

WT and Letmd1 KO mice at 4 days of age (P4) were placed individually into 6-well plates. Surface temperatures of the animals were determined with an infrared camera (FLIR). Images were analyzed using FLIR software.

#### Glucose disposal and insulin tolerance test

For the glucose tolerance test (GTT), mice fed a HFD were fasted overnight and injected with glucose (1 g/kg of body weight) intraperitoneally. The insulin tolerance test (ITT) was performed in 6 hour fasted mice by intraperitoneal injection of insulin (0.75 U/kg of body weight). Blood glucose was measured at indicated time points using a glucometer.

#### ^18^F-FDG-PET imaging and analysis

Mice were fasted overnight for 12 hours and were injected with 0.3mCi of [^18^F]Fluorodeoxy-glucose and a 15 minute static PET image was obtained 45 minutes later. Animals were anesthetized with 1.5%–2.0% isoflurane and maintained with 1.0%–1.5% isoflurane for the duration of imaging. WT and KO mouse were imaged side-by-side in one of two preclinical PET systems (Inveon Dedicated PET from Siemens or microPET Focus 120 from CTI-Concorde Microsystems LLC). PET data were reconstructed using 2 iterations of a 3-D ordered subset expectation maximization (OSEM3D) followed by 18 iterations of a maximum *a posteriori* (MAP) algorithm. The imaging matrix was 128 × 128 × 95 with reconstructed voxel sizes of 0.43 mm × 0.43 mm × 0.80 mm. Image processing and analysis were performed using PMOD v.4.102 (PMOD Technologies). A 2 mm spherical volume-of-interest (VOI) was placed over the central hotspot of BAT. The standardized uptake value (SUV) of the 8 hottest voxels in this 2 mm sphere was determined for each BAT.

#### Histology and immunohistochemistry (IHC)

BAT or liver from WT and Letmd1 KO mouse was isolated and fixed in 10% neutral buffered formalin (Fisher Scientific) overnight, dehydrated, and embedded in paraffin. The paraffin sections were stained with hematoxylin and eosin and images obtained by light microscopy (20x magnification). For IHC, paraffin sections were incubated in 10% hydrogen peroxide, hydrated, and heated at 110°C for 20 min for antigen retrieval. The tissue sections were blocked in PBS containing 10% goat serum for 20 min at RT, followed by incubation with UCP1 primary antibody (1:50; Cell Signaling #72298) overnight at RT. The sections were then incubated with biotinylated rabbit secondary antibody (1:1000) for 1 hour at RT, treated with avidin-biotin complex kit (Vector Laboratories), and developed for 1 min using DAB solution (Vector Laboratories). The slides were washed with water, dehydrated, and mounted with Cytoseal 60 (Thermo Scientific). Images were taken by Nikon Eclipse 80i under 20x magnification.

#### Ectopic overexpression of genes using lentiviruses

Lentiviruses were produced by transfecting 293FT cells with a packaging construct (psPAX2; Addgene #12260), an envelope protein-producing plasmid (pCMV-VSV-G; Addgene #8454) and a lentiviral expression construct (pLenti-CMV-Puro-DEST; Addgene #17452) containing the cDNA of interest. We collected culture medium containing lentivirus at 48 hours and 72 hours after initial transfection. After centrifugation, the viral supernatant was stored at −80°C until use. At the time of transduction, the viral supernatant was diluted 2-fold with DMEM containing 10% FBS and 8 μg/ml polybrene and added to primary BAT cells on day −3 of differentiation. On the following day (day −2), cells were replenished with fresh DMEM containing 10% FBS. When confluent, cells were differentiated with induction medium (day 0), and then replenished with maintenance medium every 2 days until cells were fully differentiated.

#### Co-immunoprecipitation mass spectrometry

SVF from BAT were isolated and cultured, and transduced with lentivirus expressing Letmd1-V5 on day −3 of differentiation. Cells were lysed on day 8 in IP lysis buffer. The lysate was immunoprecipitated with a V5 tag monoclonal antibody and the eluted samples were precipitated with methanol and chloroform, dissolved in 50 mM Tris-HCl, pH 7.5, containing 8 M urea, 50 mM EDTA, and 0.005% n-dodecyl β-d-maltoside, and digested in solution with trypsin at 37°C for 12h. Digested peptides were desalted and subjected to reverse phase liquid chromatography with tandem mass spectrometry (LC-MS/MS) with a Thermo Orbitrap Fusion Lumos Tribid mass spectrometer with electron transfer dissociation-high dynamic range (ETD HD). The obtained data were searched against the International Protein Index mouse database. Proteins were identified with at least two unique valid peptides.

#### Immunoprecipitation

Cells were lysed in immunoprecipitation (IP) lysis buffer (50 mM HEPES pH 7.4, 150 mM NaCl, 10% glycerol, 1.5 mM MgCl_2_, 1 mM EDTA, 1 mM EGTA, 1% Triton X-100, 100 mM sodium fluoride, 1 mM sodium orthovanadate, 1 × protease inhibitor and 1 × phosphatase inhibitor). The lysate was immunoprecipitated using Dynabeads protein A (ThermoFisher) with an anti-Brg1 (Cell Signaling, 1:50), anti-V5 (ThermoFisher, 1:50), anti-rabbit IgG (Cell Signaling, 1:50), or anti-mouse IgG (Cell Signaling, 1:50).

#### GST pull-down assay

Glutathione S-transferase (GST), GST-Letmd1-Flag and GST-Brg1-Flag fusion proteins, and their deletion mutants (Table S1) were expressed in *E. coli* (BL21) and purified with glutathione-agarose beads (Pierce). Detection of protein-protein interactions using the GST fusion protein pull-down technique (Sambrook and Russell, 2006). Cell lysates were prepared from HeLa cells expressing either Brg1-V5 or Letmd1-V5 proteins and precleared by incubating with glutathione-agarose beads and GST for 2 hours at 4°C and centrifuging to pellet the beads. Precleared cell lysate and glutathione-agarose beads were mixed with 10 μg of GST protein or 10 μg of GST-Letmd1-Flag or GST-Brg1-Flag or one of their deletion mutants, incubated for 6 hours at 4°C with mixing, and centrifuged for 2 min. The beads were washed four times with 1 mL of GST lysis buffer. GST fusion proteins were eluted, separated by SDS-PAGE and probed with anti-V5 or anti-Flag antibody.

#### Chromatin Immunoprecipitation (ChIP)-sequencing

BAT tissues from Letmd1 KO and WT mice were dissected and processed for ChIP-sequencing using a SimpleChIP Enzymatic Chromatin IP Kit (Cell Signaling) and anti-Brg1 (Cell Signaling). Barcode-indexed sequencing libraries were generated with the KAPA Hyper DNA Library Prep Kit (Roche, KK8504). The libraries were amplified with 14 PCR cycles and analyzed with a Bioanalyzer 2100 instrument (Agilent), quantified by fluorometry on a Qubit instrument (ThermoFisher), and combined in two pools at equimolar ratios. The pools were quantified by qPCR with a KAPA Library Quant kit (Roche) and sequenced on one lane of an Illumina HiSeq 4000 (Illumina) with single-end 100 bp reads. The ChIP-seq data were deposited to Gene Expression Omnibus under the accession number GSE163204.

#### ChIP-seq data analysis

Sequencing reads were aligned to mouse genome, mm10, using STAR aligner (Dobin et al., 2013). Only uniquely aligned reads were retained, and redundant ones were deduplicated. Peaks were called using findPeaks in Homer (Heinz et al., 2010) in histone mode to identify broadly enriched signal. Peaks that overlap with ENCODE blacklist regions (Amemiya et al., 2019) were discarded. Then, peaks were pooled and merged to prepare a master peak sets for differential analysis using bedtools (Quinlan and Hall, 2010). Differential analysis was performed using EdgeR (Robinson et al., 2010). BigWig files were created using bedGraphToBigWig (Kent et al., 2010), and their heatmap profiles were extracted and visualized using bwtool (Pohl and Beato, 2014) and R.

#### RNA-sequencing and data processing

Total RNA samples from BAT or iWAT of mice housed at 22°C or at 7°C were isolated using Trizol (ThermoFisher) and purified with DNase-treated spin column (Zymo Research). Sequencing libraries were prepared and high-throughput 3′-Tag-sequencing performed using a HiSeq 4000 system (Illumina).

The raw single-end reads were quality-checked using fastqc and trimmed to remove adaptor sequences. The processed reads were aligned to GRCm38 primary genome assembly using GENCODE v21 annotation to generate counts per gene. Differential expression analysis was conducted using the limma-voom Bioconductor pipeline (Ritchie et al., 2015). Hierarchical clustering was done using R (version 3.4.3). The RNA-seq data for BAT have been deposited in Gene Expression Omnibus repository under the accession number GSE162907. The data for iWAT is accessible under the accession number GSE133619.

#### Oxygen consumption measurements

The oxygen consumption rate (OCR) was measured using a respirometer (Strathkelvin Instruments, MT200) equipped with a Clark-type electrode (Strathkelvin Instruments, SI130) in primary BAT cells in the absence or presence of 10 μM norepinephrine. Simultaneous recordings were made with two electrodes, one for each of the two experimental groups being compared, and measurements from 5 or more runs were combined for analysis. Data were analyzed by Strathkelvin 782 system data analysis module (version 4.1).

#### Cell fractionation

BAT isolated from 12-week-old mice was used for the subcellular fractionation following published protocol (Dimauro et al., 2012). Briefly, tissues were homogenized with a Dounce homogenizer in STM buffer (250 mM Sucrose, 50 mM Tris-HCl, 5 mM MgCl_2_, 1 × protease inhibitor and 1 × phosphatase inhibitor). The homogenate was centrifuged at 800 g for 15 min at 4°C. The pellet (P_0_) was resuspended in NET buffer (20 mM HEPES, 1.5 mM MgCl_2_, 0.5 M NaCl, 0.2 mM EDTA, 20% Glycerol, 1% Triton X-100, 1 × protease inhibitor and 1 × phosphatase inhibitor), broken by syringe needle and sonication, and centrifuged at 9,000 g for 30 min at 4°C. The resulting supernatant was designated as the nuclear fraction. The mitochondrial and cytosolic fractions were isolated from the supernatant after the first centrifugation step at 800 g (S_0_). The mitochondrial fraction was pelleted from S_0_ by centrifugation at 11,000 g for 10 min and resuspended in SOL buffer (50 mM Tris-HCl, 1 mM EDTA, 0.5% Triton X-100, 1 × protease inhibitor and 1 × phosphatase inhibitor). The supernatant after the 11,000 g centrifugation step was precipitated with 100% acetone and the pellet was resuspended in STM buffer to yield the cytosolic fraction.

#### Immunofluorescence

A Letmd1 rabbit polyclonal antibody (LS Bio, dilution 1:200), Brg1 mouse monoclonal antibody (ThermoFisher, 1:100), goat anti-rabbit Alexa Fluor 488 secondary antibody (ThermoFisher, 1:500), and goat anti-mouse Alexa Flour 594 secondary antibody (ThermoFisher, 1:500) were used. Primary BAT cells were isolated from WT mice and plated on round coverslips. The following day, cells were fixed with 4% formaldehyde in PBS for 10 min, washed with PBS, permeabilized with 0.25% Triton X-100 in PBS for 10 min, washed, and blocked with 10% BSA for 30 min. Cells were then incubated in primary antibodies diluted in 1% BSA overnight at 4°C, washed 3 times with PBS, incubated in secondary antibodies diluted in 1% BSA for 2 hours at room temperature, and washed 3 times with PBS. Slides were then mounted with Vectashield antifade mounting medium (Vector Labs), coverslipped, and imaged with an Olympus FV3000 confocal laser scanning microscope.

### QUANTIFICATION AND STATISTICAL ANALYSIS

All data in this study were represented as mean ± SD unless otherwise noted. Two-tailed Student’s t test was performed to analyze the difference between two independent groups. Two-way ANOVA followed by Fisher’s LSD test was used for energy expenditure comparisons after CL treatment and for body weight comparisons over time. Multiple linear regression analysis (analysis of covariance, ANCOVA) was used to assess the impact of covariates such as body weight on energy expenditure. * p ≤ 0.05, ** p ≤ 0.01, *** p ≤ 0.001. Statistical significance was considered at p ≤ 0.05. The value of n is provided in each figure legend.

## Supplementary Material

Supplementary material

## Figures and Tables

**Table:** KEY RESOURCES TABLE

REAGENT or RESOURCE	SOURCE	IDENTIFIER
Antibodies		
Rabbit anti-LETMD1	LSBio	Cat# LS-C335200
Rabbit anti-BRG1	Bethyl	Cat# A300-813A-T; RRID:AB_2191850
Rabbit anti-BRG1	Cell Signaling	Cat# 49360S; RRID:AB_2728743
Mouse anti-BRG1	ThermoFisher	Cat# PIMA531550
Mouse anti-UCP1	R&D systems	Cat# MAB6158; RRID:AB_10572490
Rabbit anti-UCP1	Cell Signaling	Cat# 72298
Sheep anti-PRDM16	R&D Systems	Cat# AF6295; RRID:AB_10717965
Sheep anti-EBF2	R&D Systems	Cat# AF7006; RRID:AB_10972102
Mouse anti- OXPHOS	MitoSciences	Cat# MS604; RRID:AB_2629281
Mouse anti-LAMIN	Santa Cruz	Cat# sc-376248; RRID:AB_10991536
Rabbit anti-VDAC	Cell Signaling	Cat# 4661T; RRID:AB_10557420
Mouse anti-FABP4	Santa Cruz	Cat# sc-271529; RRID:AB_10650265
Mouse anti-Flag	GenScript	Cat# A00187; RRID:AB_1720813
Mouse anti-V5	ThermoFisher	Cat# R96025; RRID:AB_159313
Mouse anti-b-ACTIN	Santa Cruz	Cat# sc-47778; RRID:AB_2714189
Rabbit anti-GAPDH	Santa Cruz	Cat# sc-25778; RRID:AB_10167668
Mouse anti-VINCULIN	Sigma	Cat# V9131; RRID:AB_477629
Goat anti-mouse IgG (H+L) HRP	Jackson ImmunoResearch	Cat# 115-035-003; RRID:AB_10015289
Goat anti-rabbit IgG (H+L) HRP	Jackson ImmunoResearch	Cat# 112-035-003; RRID:AB_2338128
Donkey anti-sheep IgG (H+L) HRP	Jackson ImmunoResearch	Cat# 713-035-003; RRID:AB_2340709
Goat anti-rabbit Alexa Fluor 488 secondary antibody	ThermoFisher	Cat# A11008; RRID:AB_143165
Goat anti-mouse Alexa Fluor 594 secondary antibody	ThermoFisher	Cat# A11005; RRID:AB_141372
Biotinylated anti-rabbit IgG (H+L)	Cell Signaling	Cat#14708; RRID:AB_2798581
Chemicals, peptides, and recombinant proteins		
Collagenase D	Sigma Aldrich	Cat# 11088882001
Dispase II	Sigma Aldrich	Cat# D4693
Insulin (cell culture)	Sigma Aldrich	Cat# I0516
3-Isobutyl-1-methylxanthine	Sigma Aldrich	Cat# I5879
Dexamethasone	Sigma Aldrich	Cat# D4902
Indomethacin	Sigma Aldrich	Cat# I7378
3,3′,5-triiodo-L-thyronine	Sigma Aldrich	Cat# T6397
Rosiglitazone	Cayman Chemical	Cat# 71740
CL316243	Sigma Aldrich	Cat# C5976
Norepinephrine bitartrate	Sigma Aldrich	Cat# 489350
Insulin (insulin tolerance test)	ThermoFisher	Cat# 12585014
Critical commercial assays		
SuperScript IV reverse transcriptase	ThermoFisher	Cat# 18091200
SSoAdvanced Universal SYBR Green Supermix	Bio-Rad	Cat# 172-5272
SimpleChIP Enzymatic Chromatin IP Kit	Cell Signaling	Cat# 9005S
Deposited data		
RNA-Seq data	NCBI GEO	GSE133619
RNA-Seq data	NCBI GEO	GSE162907
ChIP-Seq data	NCBI GEO	GSE163204
Experimental models: Cell lines		
HeLa cell	ATCC	RRID: CVCL_0030
293FT cell	Invitrogen	Cat# R70007
Experimental models: Organisms/strains		
Mouse: C57BL/6J	JAX	Cat# 000664
Mouse: C57BL/6J-Letmd1^em1Mbp^	UC Davis Mouse Biology Program	N/A
Oligonucleotides		
See Table S1	This paper	N/A
Recombinant DNA		
Plasmid: psPAX2	Trono Lab	Addgene Plasmid # 12260
Plasmid: pCMV-VSV-G	(Stewart et al., 2003)	Addgene Plasmid # 8454
Plasmid: pLenti-CMV-Puro-DEST	(Campeau et al., 2009)	Addgene Plasmid # 17452
Plasmid: pGEX6P1-DEST-FLAG	Jackson and Reijns	Addgene Plasmid # 119754
Plasmid: pLenti-CMV-LacZ	This paper	N/A
Plasmid: pLenti-CMV-Letmd1-V5	This paper	N/A
Plasmid: pLenti-CMV-BRG1	This paper	N/A
Plasmid: pLenti-CMV-BRG1-V5	This paper	N/A
Software and algorithms		
GraphPad Prism version 8	GraphPad	https://www.graphpad.com/
ImageJ	NIH	https://imagej.nih.gov/ij/
R version 3	The R foundation	https://www.r-project.org/
Integrative Genomics Viewer	Broad Institute	https://software.broadinstitute.org/
Limma Bioconductor	Ritchie et al., 2015	https://bioconductor.org/packages/release/bioc/html/limma.html
STAR	Dobin et al., 2013	https://code.google.com/archive/p/rna-star/
Homer	Heinz et al., 2010	http://homer.ucsd.edu/homer/
Bedtools	Quinlan and Hall, 2010	http://code.google.com/p/bedtools
EdgeR	Robinson et al., 2010	https://bioconductor.org/packages/release/bioc/html/edgeR.html
bedGraphToBigWig	Kent et al., 2010	http://hgdownload.cse.ucsc.edu/admin/jksrc.zip
Bwtool	Pohl and Beato, 2014	http://cromatina.crg.eu/bwtool.
Other		
High fat diet	Research Diets	Cat# D12492

## Highlights

- Letmd1 is a brown-fat-enriched protein induced by cold and β-adrenergic signaling
- β3-adrenoreceptor-dependent energy expenditure in mice requires Letmd1
- Letmd1 loss causes abnormal brown fat mitochondria and thermogenic gene expression
- Letmd1 interacts with the chromatin remodeler BRG1 to regulate thermogenic genes
