# Supplementary material for: Defective brown adipose tissue thermogenesis and impaired glucose metabolism in mice lacking Letmd1
Source: Cell Rep. Author manuscript; Available in PMC 2025 Apr 17. (PMC12003058; doi:10.1016/j.celrep.2021.110104)
Supplement: Supplementary material [file NIHMS2069046-supplement-Supplementary_material.pdf]

**Cell Reports, Volume *37***

## Supplemental information

# Defective brown adipose tissue thermogenesis

# and impaired glucose metabolism

# in mice lacking Letmd1

**Kyung-Mi Choi, Jung Hak Kim, Xiangmudong Kong, Meltem Isik, Jin Zhang, Hee-Woong Lim, and John C. Yoon**

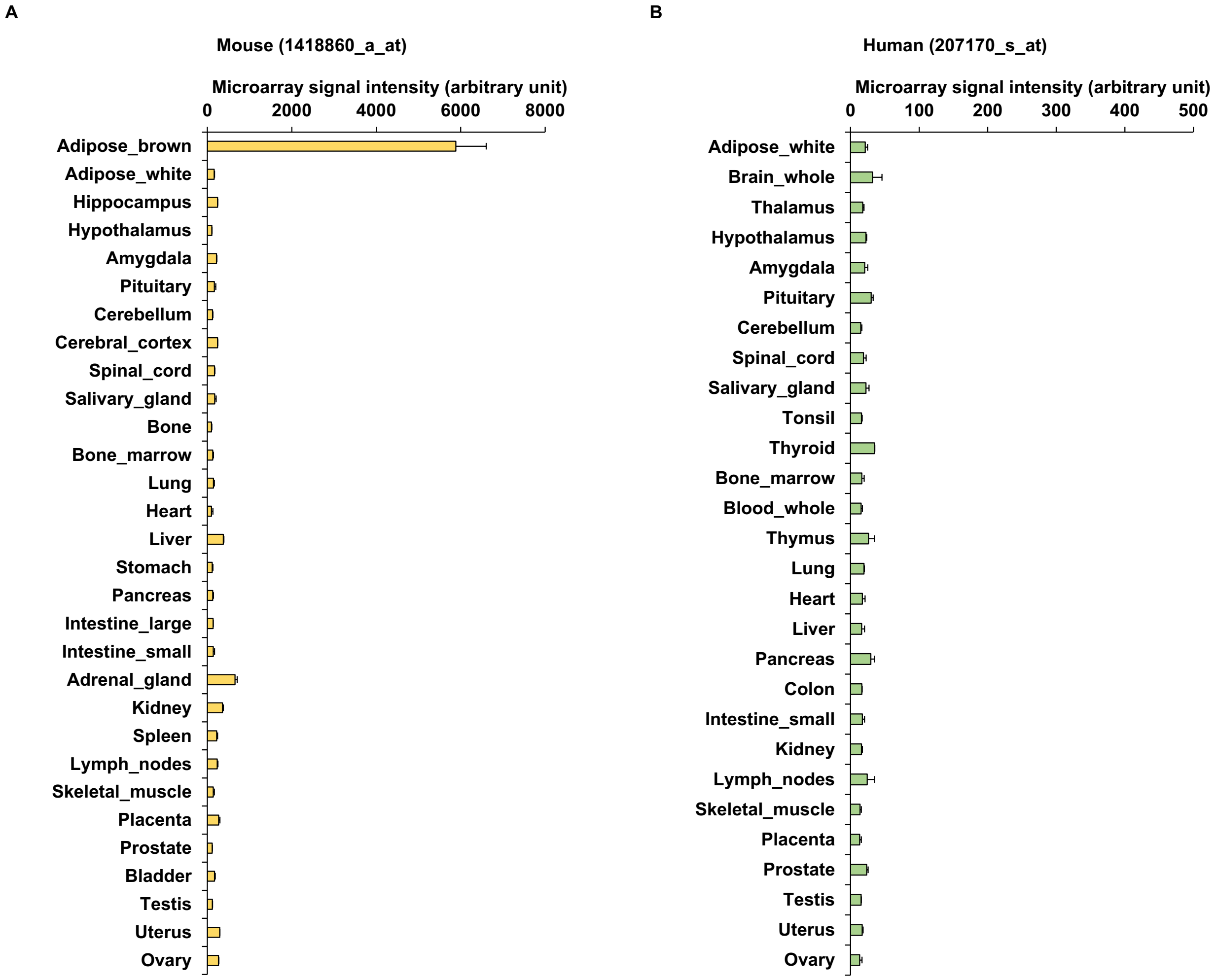


**Figure S1. Letmd1 gene expression profiles in mouse and human tissues. Related to Figure 1.**

(A) Relative gene expression in 30 mouse tissues obtained from NCBI GEO GSE10246 (1418860_a_at).

(B) Relative Letmd1 gene expression in 28 human tissues obtained from NCBI GEO GSE1133 (207170_s_at). Note that this study did not include human brown adipose tissue.

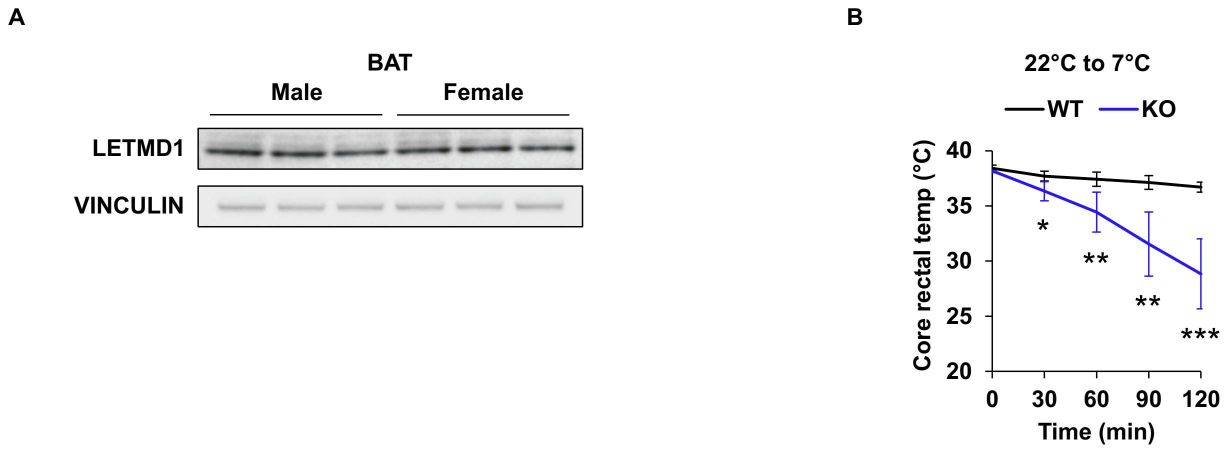


**Figure S2. Letmd1 is expressed at comparable levels in BAT from male and female mice and loss of Letmd1 causes cold intolerance in female mice. Related to Figure 2.**

(A) LETMD1 protein expression in BAT from 6-week-old male and female mice.

(B) Core rectal temperature after acute cold exposure in 10-week-old female WT and Letmd1 KO mice. WT, n = 5; KO, n = 5. Data are mean ± SD. *P*-values are calculated by Student's t-test.

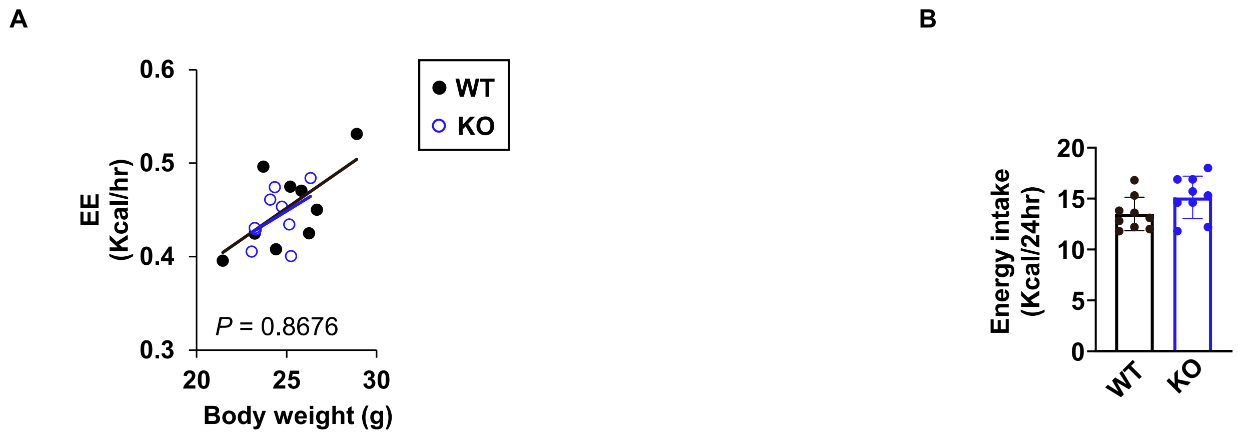


**Figure S3. Indirect calorimetry in Letmd1 KO mice. Related to Figure 2.**

(A) Average 24-hour energy expenditure in 13-week-old male Letmd1 KO (n = 9) mice and WT littermates (n = 9) on a regular diet housed at 22°C.

(B) Average 24-hour energy intake in KO (n = 9) and WT (n = 9) mice on a RD.

Data are mean ± SD. *P*-values in (A) and (B) are determined by ANCOVA and Student's t-test, respectively.

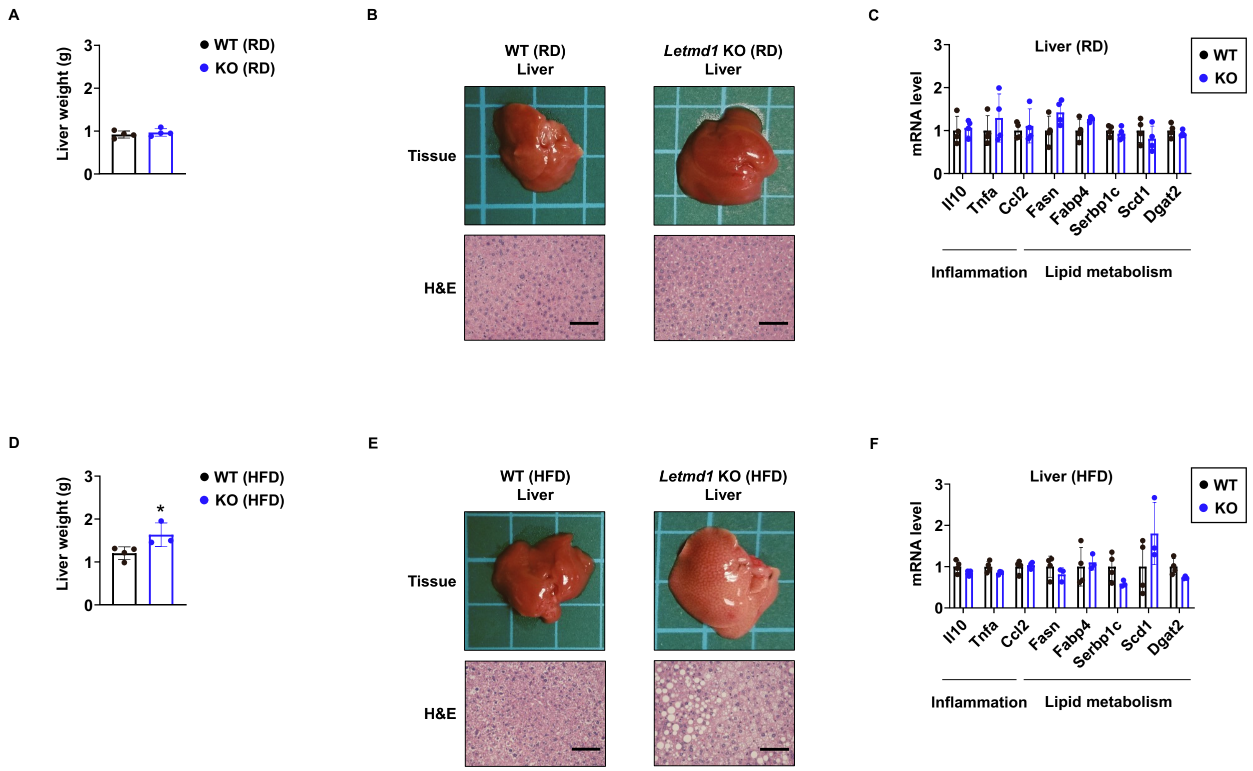


**Figure S4. Livers from the Letmd1 KO mice are similar to WT mice on a regular diet but accumulate more lipid on a HFD. Related to Figure 3.**

(A) Liver weights of 21-week-old male WT and Letmd1 KO mice fed a RD. n = 4 for each group.

(B) Gross tissue image and H&E staining of liver from WT and Letmd1 KO mice on a chow diet. Scale bar = 100 µm.

(C) mRNA expression of genes related with inflammation and lipid metabolism in liver from WT and Letmd1 KO mice on a chow diet. n = 4 for each group.

(D to F) Liver weight (D), tissue image and H&E (E), and gene expression (F) from 22-week-old male WT and Letmd1 KO mice on a HFD. n = 4 for each group. Scale bar = 100 µm.

Bar graphs are mean ± SD. *P*-values are calculated by Student's t-test.

**Table S1. List of PCR primers used in this study. Related to STAR Methods section.**

| Primer | Forward (5' to 3') | Reverse (5' to 3') | Purpose |
|---|---|---|---|
| Letmd1 Genotyping | GTTTACCTAGAGATCCCGATAAAAGGC | GACTGAAGCAGGAAGTTTGATGACC | Genotyping of Letmd1 KO |
| Letmd1 WT | ATGGCGCTGTCCAGGGTGTG | TCAGCGCCTTGTCTCAAGGTAGTTGGT | Letmd1 cloning |
| Letmd1-V5 | ATGGCGCTGTCCAGGGTGTG | GCGCCTTGTCTCAAGGTAGTTGGTG | Letmd1-V5 cloning |
| Brg1-V5 | GCCACCATGTCTACTCCAGACCCAC | GTCTTCCTCACTGCCACTTCCTG | Brg1-V5 cloning |
| Mito DNA (MT-ND1) | CTAGCAGAAACAAACCGGGC | CCGGCTGCGTATTCTACGTT | Quantification of mito DNA |
| Nuclear DNA (HK2) | GCCAGCCTCTCCTGATTTTAGTGT | GGGAACACAAAAGACCTCTTCTGG | Quantification of nuclear DNA |
| Letmd1 | CTACCCACATTGCTGACGAC | GGGACAGCTCGGGTTCTT | qPCR |
| Ppargc1a | GAAAGGGCCAAACAGAGAGA | GTAAATCACACGGCGCTCTT | qPCR |
| Prdm16 | CAGCACGGTGAAGCCATTC | GCGTGCATCCGCTTGTG | qPCR |
| Ucp1 | ACTGCCACACCTCCAGTCATT | CTTTGCCTCACTCAGGATTGG | qPCR |
| Cidea | TGCTCTTCTGTATCGCCCAGT | GCCGTGTTAAGGAATCTGCTG | qPCR |
| Cox5a | GGGTCACACGAGACAGATGA | GGAACCAGATCATAGCCAACA | qPCR |
| Dio2 | CAGTGTGGTGCACGTCTCCAATC | TGAACCAAAGTTGACCACCAG | qPCR |
| Fabp4 | AAGGTGAAGAGCATCATAACCCT | TCACGCCTTTCATAACACATTCC | qPCR |
| Pparg | GAAAGACAACGGACAAATCACC | GGGGGTGATATGTTTGAACTTG | qPCR |
| Il10 | GCTCTTACTGACTGGCATGAG | CGCAGCTCTAGGAGCATGTG | qPCR |
| Tnfa | ATGGCCTCCCTCTCATCAGT | TTTGCTACGACGTGGGCTAC | qPCR |
| Ccl2 | AGGTCCCATGTCATGCTTCTGG | CTGCTGCTGGTGATCCTCTTG | qPCR |
| Fasn | AGTTCACGGACATGGAGCACAACA | ATGGTACTTGGCCTTGGGTGTGTA | qPCR |
| Serbp1c | ATCGGCGCGGAAGCTGTCGG | GGGAAGTCACTGTCTTGGTTG | qPCR |
| Scd1 | GCTGGAGTACGTCTGGAGGAA | TCCCGAAGAGGCAGGTGTAG | qPCR |
| Dgat2 | TACTCCAAGCCCATCACCAC | CAGTTCACCTCCAGCACCTC | qPCR |
| β-Actin | CTAAGGCCAACCGTGAAAAG | ACCAGAGGCATACAGGGACA | qPCR (normalization control) |

**Table S1. List of PCR primers used in this study (continued).**

| Oligonucleotide for GST-Pull down | Forward (5' to 3') | Reverse (5' to 3') | Purpose |
|---|---|---|---|
| Brg1 FL (1-1647) | GCCACCATGTCTACTCCAGACCCAC | GTCTTCCTCACTGCCACTTCCTG | Brg1 GST-pull down |
| Brg1 Del-1 (1-1433) | GCCACCATGTCTACTCCAGACCCAC | CTTGTCACGGCTGCGGGTG | Brg1 GST-pull down |
| Brg1 Del-2 (1-700) | GCCACCATGTCTACTCCAGACCCAC | CTCAGAGACATCATCGCTGTCTGG | Brg1 GST-pull down |
| Brg1 Del-3 (1-550) | GCCACCATGTCTACTCCAGACCCAC | CAGGCGCTTGTCCTTCTTCTGG | Brg1 GST-pull down |
| Brg1 Del-4 (1-330) | GCCACCATGTCTACTCCAGACCCAC | TGTTTGTGGTGGCATTACAGGTGAG | Brg1 GST-pull down |
| Brg1 Del-5 (701-1647) | GTGGACGCCCGACACATTATT | same as Brg1 FL, reverse | Brg1 GST-pull down |
| Brg1 Del-6 (701-1433) | same as Del-5 | same as Brg1 Del-1, reverse | Brg1 GST-pull down |
| Brg1 Del-7 (1428-1647) | CACCCGCAGCCGTGACAAG | same as Brg1 FL, reverse | Brg1 GST-pull down |
| Letmd1 FL (1-360) | GCCACCATGTCTACTCCAGACCCAC | TCAGCGCCTTGTCTCAAGGTAGTTGGT | Letmd1 GST-pull down |
| Letmd1 Del-1 (1-340) | GCCACCATGTCTACTCCAGACCCAC | CTCGGGTTCTTTCAGGCTACAGGAG | Letmd1 GST-pull down |
| Letmd1 Del-2 (1-165) | GCCACCATGTCTACTCCAGACCCAC | CAGTTGCCTAGGAAACAGGTAC | Letmd1 GST-pull down |
| Letmd1 Del-3 (1-120) | GCCACCATGTCTACTCCAGACCCAC | GAGTTGATGAAACTTTAGGTTTTGTTTCCACA | Letmd1 GST-pull down |
| Letmd1 Del-4 (166-360) | CTAGTCAAGCATTTCTGGACCCCC | same as Letmd1 FL, reverse | Letmd1 GST-pull down |

**Table S2. List of genes enriched in mouse BAT versus WAT, human BAT versus WAT, and mouse beige fat versus WAT (top 30 based on fold-change in mouse beige fat). Related to Figure 1.**

| MGI_symbol | Full name |
|---|---|
| Ucp1 | uncoupling protein 1 (mitochondrial, proton carrier) |
| Pank1 | pantothenate kinase 1 |
| Hadha | hydroxyacyl-CoA dehydrogenase trifunctional multienzyme complex subunit alpha |
| Gk | glycerol kinase |
| Letmd1 | LETM1 domain containing 1 |
| Acadvl | acyl-Coenzyme A dehydrogenase, very long chain |
| Ciapin1 | cytokine induced apoptosis inhibitor 1 |
| Poln | DNA polymerase N |
| Acsl5 | acyl-CoA synthetase long-chain family member 5 |
| Suclg1 | succinate-CoA ligase, GDP-forming, alpha subunit |
| Ndufv1 | NADH:ubiquinone oxidoreductase core subunit V1 |
| Acsf2 | acyl-CoA synthetase family member 2 |
| Pdha1 | pyruvate dehydrogenase E1 alpha 1 |
| Uqcr10 | ubiquinol-cytochrome c reductase, complex III subunit X |
| Vwa8 | von Willebrand factor A domain containing 8 |
| Ndufs8 | NADH:ubiquinone oxidoreductase core subunit S8 |
| Cox5a | cytochrome c oxidase subunit 5A |
| Kcnk3 | potassium channel, subfamily K, member 3 |
| Idh3b | isocitrate dehydrogenase 3 (NAD+) beta |
| mt-Nd2 | mitochondrially encoded NADH dehydrogenase 2 |
| Ehhadh | enoyl-Coenzyme A, hydratase/3-hydroxyacyl Coenzyme A dehydrogenase |
| Idh3a | isocitrate dehydrogenase 3 (NAD+) alpha |
| Aco2 | aconitase 2, mitochondrial |
| Ppif | peptidylprolyl isomerase F (cyclophilin F) |
| Ndufb9 | NADH:ubiquinone oxidoreductase subunit B9 |
| Cox10 | heme A:farnesyltransferase cytochrome c oxidase assembly factor 10 |
| Ptcd3 | pentatricopeptide repeat domain 3 |
| Sod2 | superoxide dismutase 2, mitochondrial |
| Ndufab1 | NADH:ubiquinone oxidoreductase subunit AB1 |
| Ndufs2 | NADH:ubiquinone oxidoreductase core subunit S2 |

**Table S3. List of Letmd1-interacting proteins related to gene expression detected by IP mass spectrometry. Related to Figure 5.**

| UNIPROT ID | Protein |
|---|---|
| P43274 | H1.4 linker histone, cluster member |
| P43277 | H1.3 linker histone, cluster member |
| P15864 | H1.2 linker histone, cluster member |
| Q61656 | DEAD box helicase 5 |
| E9QAT0 | FMRP translational regulator 1 |
| A2AFK7 | RNA helicase |
| Q8R081 | heterogeneous nuclear ribonucleoprotein L |
| P62960 | Y box protein 1 |
| P61979 | heterogeneous nuclear ribonucleoprotein K |
| P29341 | poly(A) binding protein, cytoplasmic 1 |
| Q9D0E1 | heterogeneous nuclear ribonucleoprotein M |
| Q9Z2X1 | heterogeneous nuclear ribonucleoprotein F |
| P63017 | heat shock protein 8 |
| P80315 | chaperonin containing Tcp1, subunit 4 (delta) |
| P42932 | chaperonin containing Tcp1, subunit 8 (theta) |
| P80317 | chaperonin containing Tcp1, subunit 6a (zeta) |
| Q61937 | nucleophosmin 1 |
| P62806 | H4 clustered histone 1 |
| P10922 | H1.0 linker histone |
| Q3TKT4 | SWI/SNF related, matrix associated, actin dependent regulator of chromatin, subfamily a, member 4 |
| P10853 | Histone H2B type 1-F/J/L |
| P09405 | nucleolin |
